# HIV-1 mutants that escape the cytotoxic T-lymphocytes are defective in viral DNA integration

**DOI:** 10.1093/pnasnexus/pgac064

**Published:** 2022-05-20

**Authors:** Muthukumar Balasubramaniam, Benem-Orom Davids, Alex Bryer, Chaoyi Xu, Santosh Thapa, Jiong Shi, Christopher Aiken, Jui Pandhare, Juan R Perilla, Chandravanu Dash

**Affiliations:** The Center for AIDS Health Disparities Research, Meharry Medical College, Nashville, TN – 37208, USA; Department of Biochemistry, Cancer Biology, Neuroscience and Pharmacology, Meharry Medical College, Nashville, TN – 37208, USA; The Center for AIDS Health Disparities Research, Meharry Medical College, Nashville, TN – 37208, USA; Department of Biochemistry, Cancer Biology, Neuroscience and Pharmacology, Meharry Medical College, Nashville, TN – 37208, USA; School of Graduate Studies and Research, Meharry Medical College, Nashville, TN – 37208, USA; Department of Chemistry, University of Delaware, Newark, DE – 19716, USA; Department of Chemistry, University of Delaware, Newark, DE – 19716, USA; The Center for AIDS Health Disparities Research, Meharry Medical College, Nashville, TN – 37208, USA; Department of Biochemistry, Cancer Biology, Neuroscience and Pharmacology, Meharry Medical College, Nashville, TN – 37208, USA; Department of Pathology, Microbiology and Immunology, Vanderbilt University Medical Center, Nashville, TN – 37232, USA; Department of Pathology, Microbiology and Immunology, Vanderbilt University Medical Center, Nashville, TN – 37232, USA; The Center for AIDS Health Disparities Research, Meharry Medical College, Nashville, TN – 37208, USA; School of Graduate Studies and Research, Meharry Medical College, Nashville, TN – 37208, USA; Department of Microbiology, Immunology, and Physiology, Meharry Medical College, Nashville, TN – 37208, USA; Department of Chemistry, University of Delaware, Newark, DE – 19716, USA; The Center for AIDS Health Disparities Research, Meharry Medical College, Nashville, TN – 37208, USA; Department of Biochemistry, Cancer Biology, Neuroscience and Pharmacology, Meharry Medical College, Nashville, TN – 37208, USA

**Keywords:** human immunodeficiency virus (HIV), cytotoxic T lymphocytes (CTL), capsid, integration, reverse transcription

## Abstract

HIV-1 replication is durably controlled without antiretroviral therapy (ART) in certain infected individuals called elite controllers (ECs). These individuals express specific human leukocyte antigens (HLA) that tag HIV-infected cells for elimination by presenting viral epitopes to CD8+ cytotoxic T-lymphocytes (CTL). In HIV-infected individuals expressing HLA-B27, CTLs primarily target the viral capsid protein (CA)-derived KK10 epitope. While selection of CA mutation R264K helps HIV-1 escape this potent CTL response, the accompanying fitness cost severely diminishes virus infectivity. Interestingly, selection of a compensatory CA mutation S173A restores HIV-1 replication. However, the molecular mechanism(s) underlying HIV-1 escape from this ART-free virus control by CTLs is not fully understood. Here, we report that the R264K mutation-associated infectivity defect arises primarily from impaired HIV-1 DNA integration, which is restored by the S173A mutation. Unexpectedly, the integration defect of the R264K variant was also restored upon depletion of the host cyclophilin A. These findings reveal a nuclear crosstalk between CA and HIV-1 integration as well as identify a previously unknown role of cyclophilin A in viral DNA integration. Finally, our study identifies a novel immune escape mechanism of an HIV-1 variant escaping a CA-directed CTL response.

Significance Statement HIV-1 replication is durably suppressed in a small percentage of infected individuals without ART. This natural control is associated with the expression of specific HLAs that displays a fragment of the HIV-1 CA protein on the infected cells and marks them for destruction by the killer T cells. Specific CA mutations allows HIV-infected cells to escape from these killer T cells but causes severe infectivity defect. We report that this infectivity defect arises from the inability of HIV-1 mutants to integrate into human chromosomes. We also discovered a novel role of cyclophilin A in the integration of HIV-1 CTL escape mutant. Collectively, these findings significantly advance our understanding of CA function and the mechanism by which HIV-1 escapes CTL response.

## Introduction

The host immune system can control HIV and delay disease progression (1–3). For instance, the virus replication is durably suppressed and disease progression is delayed in untreated HIV-1-infected individuals known as “elite controllers-ECs” (4). Such long-term asymptomatic infection in the absence of antiretroviral therapy (ART) has been attributed to immunological mechanisms coordinated by human leukocyte antigens (HLA) (5) and CD8+ cytotoxic T-lymphocytes (CTLs) (6, 7). Notably, a strong association between virus control and presentation of viral epitopes by HLA-B57 or HLA-B27 to HIV-1-specific CTLs has been well-documented (8–11). However, CTL-mediated HIV-1 control is compromised by escape mutations that diminish recognition of viral epitopes (12–15).

HLA-B27 targets the KK10 epitope (16) located in the capsid (CA) domain of the HIV-1 Gag polyprotein (1, 17–19). However, mutations in this epitope results in the escape of HIV-1 from this immunological constraint (18, 20–23). Specifically, the prerequisite L268M (LM) mutation emerges early followed by the consequential R264K (RK) mutation selected late in the course of infection (12, 18, 24). The RK mutation inhibits viral epitope presentation (18, 25) but significantly reduces HIV-1 infectivity (12, 14, 18, 21, 24–27). This is because CA is genetically fragile (28, 29) and is a key regulator of HIV-1 replication (30–36). Particularly, CA forms the capsid shell that houses the viral genome and other viral/cellular factors (37–40). CA also provides binding interfaces for host factors like cyclophilin A (CypA) (41, 42) and cleavage and polyadenylation-specific factor 6 (CPSF6) (43, 44) to promote infection (45–49). Remarkably, the infectivity defect of the RK mutant is restored by the compensatory CA mutation S173A (SA) (21) or upon depletion of CypA (21, 26). However, the mechanism by which these CTL-escape CA mutations reduce or restore HIV-1 replication is poorly understood.

The infectivity defect of the RK mutant has been attributed to a block at the reverse transcription step (21). In CEM cells, reverse transcription of VSVg-pseudotyped RK mutant was reduced to a log-fold compared to the wild type (WT) virus. Strikingly, the reverse transcription levels of the WT and the RKLMSA viruses were comparable. Interestingly, blocking the interaction between HIV-1 CA and CypA restored the infectivity of the RK mutant (21). These data suggested that the interaction between the RK mutant capsid and CypA impaired virus replication. Additionally, data from cyclosporine washout assays in Owl monkey cells inoculated with VSVg-pseudotyped viruses, suggested that the RK mutation increased capsid stability, that was corrected by the compensatory SA mutation (27). Although, capsid stability is coupled with HIV-1 reverse transcription, the mechanism of infectivity defect remained unclear in these studies.

Here, we report the comprehensive biology of the CTL-escape RK mutant virus using a multipronged approach that included structure-guided molecular dynamics (MD) simulations, protein interaction studies by yeast two hybrid (Y2H) assay, virus infectivity assays, biochemical characterization of isolated viral replication complexes, and genetic analysis of the viral genome. Collectively, our results have uncovered that reduced HIV-1 integration is the principal mechanism underlying the infectivity defect of the RK variant. These findings also support a regulatory role of CA and a novel role of CypA in postnuclear entry steps of HIV-1 infection.

## Results

### CTL escape-associated CA mutations do not alter capsid structure

**Fig. 1.**
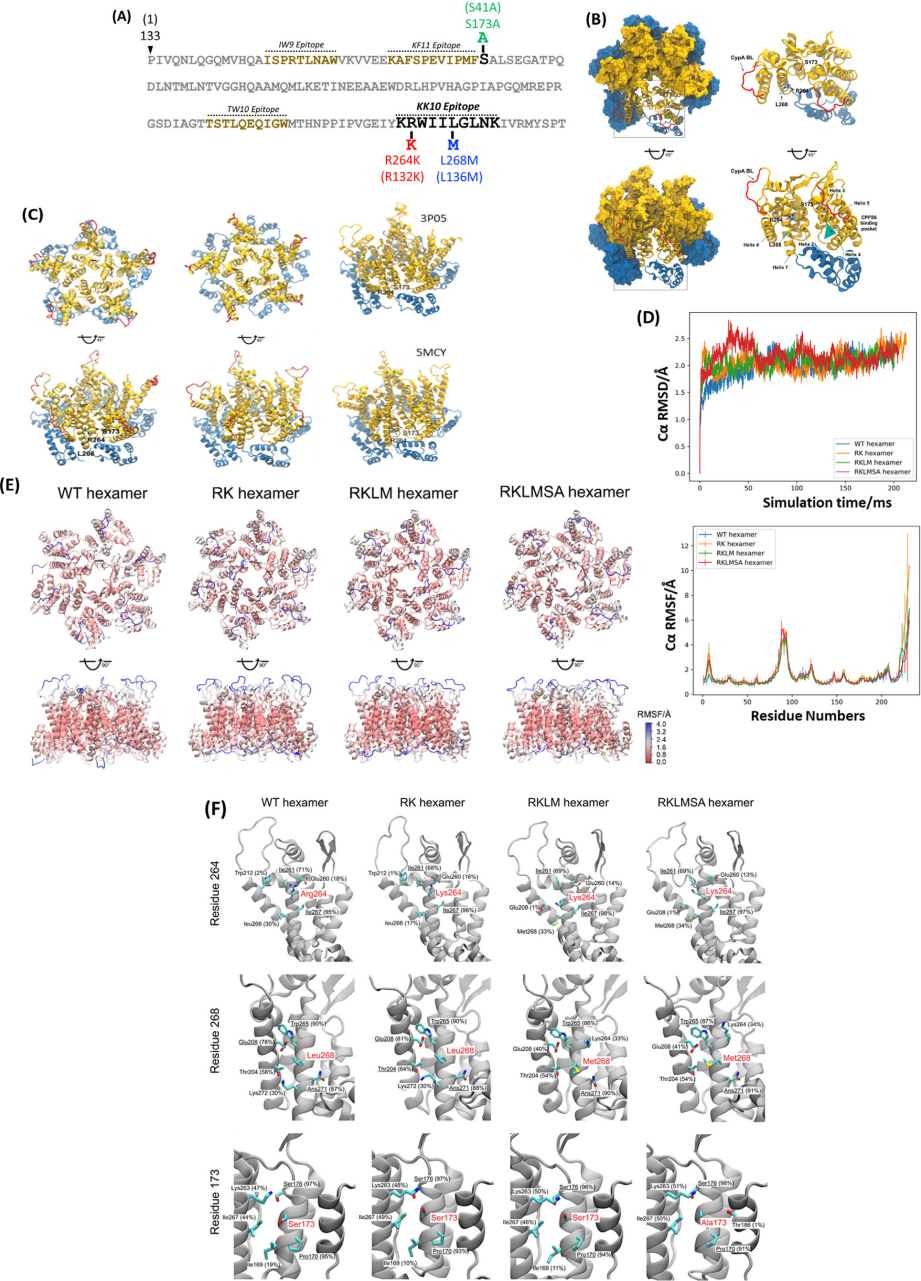
**(A)** Location of CTL-targeted epitopes in the HIV-1 CA N-terminal domain (NTD) and the CTL escape-associated amino acid changes linked to the HLA-B27-restricted KK10 epitope. The standard Gag numbering of the amino acids is followed, and the capsid numbering of the amino acids are in parentheses. **(B)** Relative positions of three CA residues, R264(R132), L268(L136), and S173(S41) in WT CA monomer and hexamer. (Left) A CA hexamer (PDBid: 4XFX) is represented by a surface model. The NTD is colored in gold, while the C-terminal domain (CTD) is in blue. (Right) Ribbon representation a CA monomer and the NTD from one of its neighboring monomers. The positions of R264, L268, and S173 residues in the NTD–NTD interface are labeled, CypA BL is colored in red, and the CPSF6 binding pocket is depicted as a green triangle. **(C)** Relative positions of R264(R132), L268(L136), and S173(S41) in a WT CA pentamer. (Left) Ribbon representation of a CA pentamer. The NTD is colored in gold, while the CTD is in blue. The positions of R264, L268, and S173 residues in the NTD–NTD interface are labeled and the CypA BL is colored in red. (Right) R264 and S173 residues on two CA pentamer models, PDB accession codes 3P05 and 5MCY. **(D)** Structural stability of HIV-1 CA hexamers. (Top) The average Cα root mean square deviations (RMSDs) of the HIV-1 WT, RK, RKLM, and RKLMSA hexamers derived from MDs simulations. (Bottom) The Cα root mean square fluctuations (RMSFs) of HIV-1 WT and mutant hexamers and from MDs simulations. **(E)** Structural flexibilities of the WT and mutant hexamers, colored by the average Cα RMSF values, increasing from red to blue. **(F)** Interhexamer interactions of three CA residues, R264(R132), L268(L136), and S173(S41). Top five interhexamer contacts involved in these CA residues are indicated. The residue names with occupancies greater than 60% are underlined. The formation of a residue contact is defined as the distance between sidechains from neighboring residues within 3.0 Å.

The conical HIV-1 capsid is assembled by ∼1,500 copies of CA monomers organized into a lattice of hexameric and pentameric units (37, 38, 50). Since the capsid structure is essential for HIV-1 infection (51), we probed whether CTL escape-associated CA mutations alter stability of the CA hexamers and tubular assemblies by molecular modeling and MD simulations. Figure 1(A) depicts the relative positions of the CTL escape-associated amino acids [R264(R132), L268(L136), and S173A(S41)] and the substitution changes [R264K, L268M, and S173A] linked to the HLA-B27-restricted KK10 epitope in the CA. These amino acids are located in the NTD–NTD interface between two neighboring CA monomers and are positioned on the exterior of the CA hexamers (Fig. 1B) and pentamers (Fig. 1C). Notably, these amino acids are not positioned in the proximity of CypA binding loop (BL) or the CPSF6 binding pocket.

MD simulations showed similar dynamic properties of the WT and CTL escape mutant hexamers (Fig. 1D–F). Specifically, the Cα root mean square deviations (RMSDs) of all the CA hexamer systems showed an increase at 20 ns and were plateaued at around 2 Å after 50 ns (Fig. 1D, top panel). Importantly, the mean Cα root mean square fluctuations (RMSFs) of all systems exhibited negligible differences, especially in the structured regions (Fig. 1D, bottom panel), and the structural flexibilities of all the hexamer systems were comparable (Fig.   1E). The interhexamer interactions of the amino acid residues 264,268, and 173 in the WT and the mutant hexamers were also maintained (Fig. 1F). For example, the top two contacts made by R264 (with residues I261 and I267), by L268 (with residues W265 and N271), and by S173 (with residues S176 and P170) are preserved in mutant hexamers. The overall electrostatic potentials of all the mutant hexamers are very similar to the WT hexamer (Figure S1, Supplementary Material) albeit the RK mutation rendering a relatively more positive potential to the sidechain. Comparison of ion occupancy of WT hexamers to that of the RK, RKLM, and RKLMSA hexamers, showed no significant difference in the interaction between the capsid and the ionic environment (Figure S2, Supplementary Material).

**Fig. 2.**
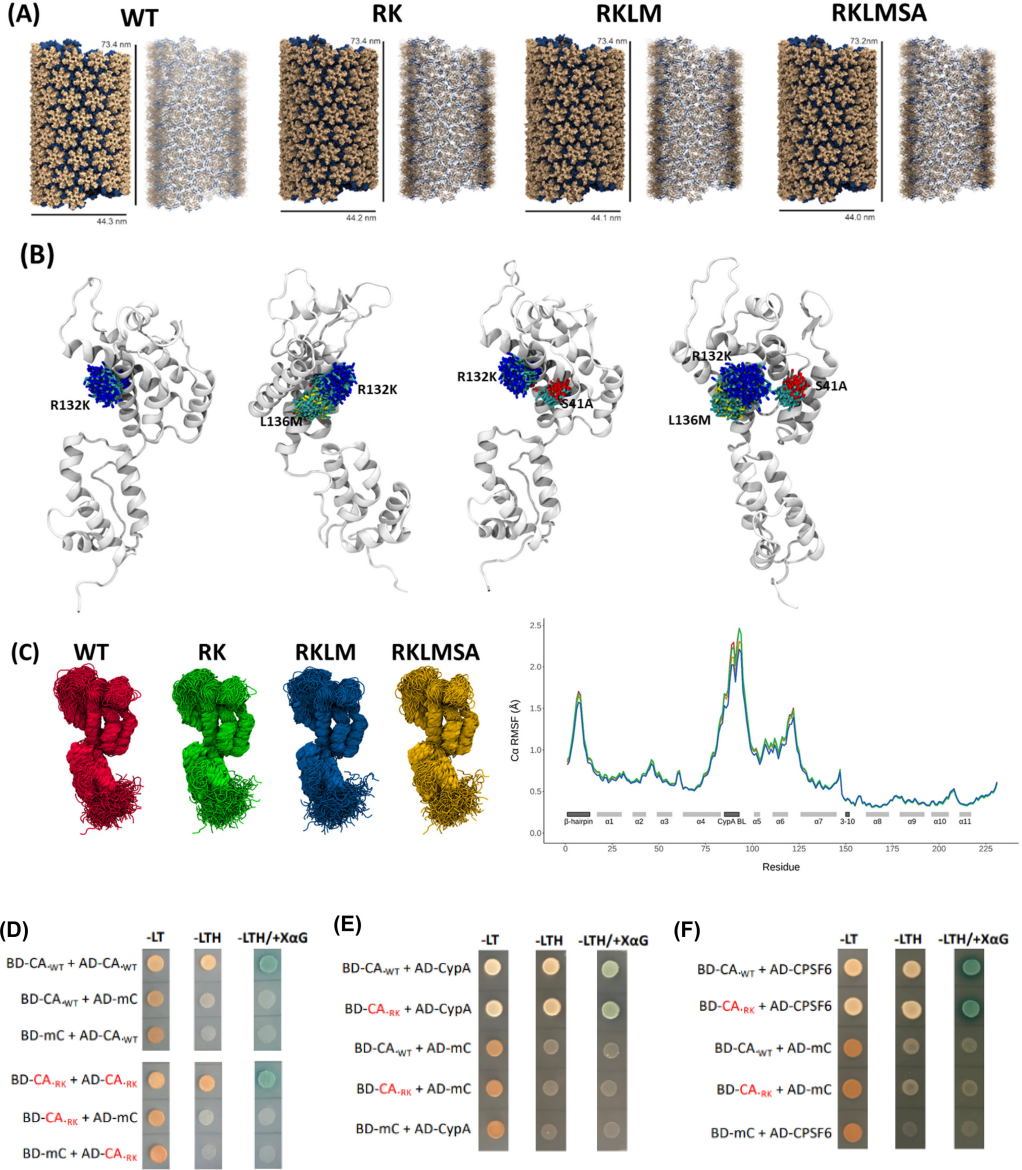
**(A)** Effects of the CTL-escape mutation on CA tube assembly. **(B)** Effects of the CTL-escape mutation on conformational distribution of CA monomers. Each mutation site, in each system, aligned to a single monomer, demonstrating the conformational distributions of these residues. **(C)** Effects of the CTL-escape mutation on the RMSF of capsid residues. (Left panel) Each monomer is aligned by backbone regions in helices. (Right panel) RMSF of carbon alpha atoms in each CA tube system. RMSF values are averaged for all monomers. **(D)–(F)** Effect of KK10-linked CTL escape mutation R264K on CA interactions by Y2H. The effect of RK mutation on **(D)** CA: CA, **(E)** CA: CypA, and **(F)** CA: CPSF6 interactions were assessed. Equal number of yeast cells cotransformed with the plasmid pairs encoding the GAL4 BD in fusion with the bait protein and the GAL4 AD in fusion with the prey protein were spotted on synthetic dropout (SD) agar plates lacking leucine and tryptophan (-LT) or leucine, tryptophan, and histidine (-LTH) or leucine, tryptophan, and histidine but supplemented with X-α-D-galactoside (-LTH/+XαG) and were incubated at 30°C to assess for histidine prototrophy and α-galactosidase activity. **(D)** Growth of yeast coexpressing BD-CA_WT_ and AD-CA_WT_, BD-CA_WT_ and AD-mCherry (AD-mC), and AD-CA_WT_ and BD-mC on selection media. (Bottom panel) Growth of yeast coexpressing BD-CA_RK_ and AD-CA_RK_, BD-CA_RK_ and AD-mC, and AD-CA_RK_ and BD-mC on selection media. **(E)** Growth of yeast coexpressing BD-CA_WT_ and AD-CypA, BD-CA_RK_ and AD-CypA, BD-CA_WT_ and AD-mC, BD-CA_RK_ and AD-mC, and BD-mC and AD-CypA on selection media. **(F)** Growth of yeast coexpressing BD-CA_WT_ and AD-CPSF6, BD-CA_RK_ and AD-CPSF6, BD-CA_WT_ and AD-mC, BD-CA_RK_ and AD-mC, and BD-mC and AD-CPSF6 on selection media.

Next, we constructed and simulated RK, RKLM, and RKLMSA mutant CA tubes to test the effects of the CA mutations on the structure and dynamics of the tubular CA assembly (Fig. 2). After 20 ns of MD equilibration, no structural differences between any of the CA tubes were observed. Tube radii and length values vary by no more than 0.1 to 0.2 nm for all mutant systems (Fig. 2A) and the conformational distributions of each mutation site were similar (Fig. 2B). Similarly, the RMSF of capsid residues remained comparable (Fig. 2C). Together, these data from molecular modeling and MD simulations illustrate that the CTL escape-associated CA amino acid changes linked in the KK10 epitope do not alter the structural integrity and dynamics of the CA hexamers and capsid lattice.

### CA mutation RK does not perturb CA–CA and CA–host protein interactions

The HIV-1 capsid structure possesses critical binding interfaces for host factor recognitions (32, 52). For example, CypA selectively bridges two neighboring CA hexamers through the CypA BL (42, 53). The CPSF6 (44) and Nucleoporin 153 (NUP153) (54) target the CA NTD–NTD interface. The central pore of CA hexamer recognizes IP6 (55), nucleotide (56), and Fasciculation and Elongation Protein Zeta 1 (FEZ1) (57) and the trihexamer region recognizes MX dynamin like GTPase 2 (MXB) (58). Since these binding interfaces are critical for HIV-1 infection, we assessed the effect of the RK mutation on CA–CA and CA–host factor interactions by yeast GAL4-based two-hybrid (Y2H) assay (59). We chose the RK mutation because it is the primary determinant of the infectivity defect associated with HLA-B27 CTL escape (21). The yeast cells cotransformed with the plasmid pairs encoding the GAL4 DNA Binding Domain fused with the WT CA (BD-CA_WT_) and the GAL4 Activation Domain in fusion with the WT CA (AD-CA_WT_), exhibited histidine prototrophy and α-galactosidase activity (Fig. 2D, top panel). Yeast cells cotransformed with plasmid pairs encoding BD-CA_WT_ and AD-mCherry or AD-CA_WT_ and BD-mCherry exhibited auxotrophy on selection media lacking leucine and tryptophan but did not exhibit histidine prototrophy or α-galactosidase activity. Importantly, we observed histidine prototrophy and α-galactosidase activity by the yeast coexpressing BD-CA_RK_ and AD-CA_RK_, but not by the negative control yeast coexpressing BD-CA_RK_ and AD-mCherry or AD-CA_RK_ and BD-mCherry (Fig. 2D, bottom panel). These results indicate that the RK mutation does not disrupt CA–CA binary interaction. Interestingly, histidine prototrophy and α-galactosidase activity was also observed in the yeast coexpressing BD-CA_WT_ or BD-CA_RK_ and AD-CypA (Fig. 2E) or AD-CPSF6 (Fig. 2F). These results demonstrate that the RK mutation has no significant effect on the binary interactions between CA and CypA or between CA and CPSF6. Together, these data strengthen our in silico studies and establish that the RK mutation minimally disrupts the structural integrity of CA or its functional interactions with key host factors.

### RK mutation impairs HIV-1 infection but the compensatory mutation SA restores infectivity

**Fig. 3.**
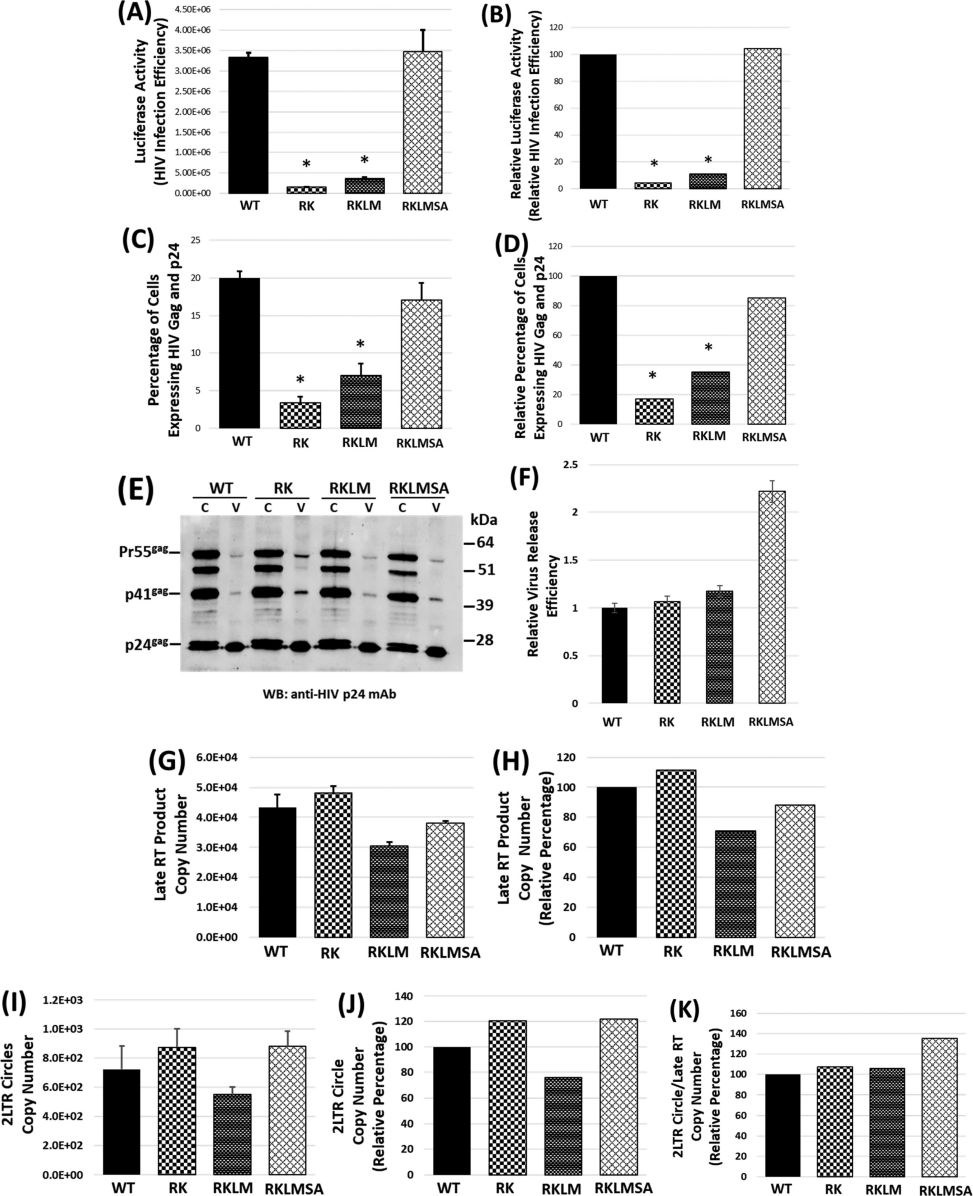
**(A)**–**(D)** Effect of KK10-linked CTL escape mutations on HIV-1 infectivity. Infectivity of WT and mutant viruses were determined using the **(A)**and**(B)** TZM-bl indicator cell line and **(C)**and**(D)** Jurkat T-cells. TZM-bl cells inoculated, in replicates, with the WT or mutant viruses for 2 hours were further cultured for 48 hours and then lysed. **(A)** Luciferase activity in the cell lysates, indicative of virus infectivity, was measured as luminescence and, after subtracting the luciferase activity in mock-infected cells from the data, were plotted as relative light units (RLU). **(B)** Relative luciferase activity, indicative of percentage infection efficiency, was plotted relative to WT. **(C)**and**(D)** Jurkat cells spinoculated, in replicates, with the WT or mutant viruses for 2 hours at 37°C were further cultured for 48 hours at 37°C, after which the cells were fixed and stained with FITC-labeled antibody that identifies viral Gag and p24. **(C)** The percentage of FITC-labeled cells was determined using FACS analysis. **(D)** Relative Gag and p24 expression was plotted relative to WT. **(E)**and**(F)** Effect of RK mutations on late stages of HIV-1 replication. HEK293T cells transfected with WT or the mutant proviral clones were cultured for 48 hours at 37°C and the viral particles in the culture media was collected by ultracentrifugation. **(E)** Cell (C) and virus (V) lysates were resolved by SDS-PAGE and the viral proteins were probed by western blotting. **(F)** The virus release efficiency was determined as described in Materials and Methods. **(G)**–**(K)** Effect of KK10-linked CTL escape mutations on HIV-1 reverse transcription and nuclear import. Copy number of late reverse transcription products and 2-LTR circles present in the total DNA isolated from Jurkat cells. **(G)** Copy number of late reverse transcription products of WT or the mutant viruses as measured by qPCR. **(H)** Percentage late RTN products plotted relative to WT. **(I)** Copy number of 2-LTR circles as measured by qPCR. **(J)** Percentage copy number of the 2-LTR circles plotted relative to WT. **(K)** Calculated ratio of the copy number of 2-LTR circles to the respective late reverse transcription products plotted as percentage relative to WT. Data are representative of three independent experiments, with error bars representing the SEMs. * represents *P* < 0.05.

HIV-1 escape from the KK10 epitope-targeted CTL response is linked to specific CA mutations (18, 20–22, 60). The prerequisite LM mutation has no significant effect on HLA binding, CTL recognition, and viral infectivity (21, 61). However, the RK mutation, independently or with LM (RKLM) significantly reduces the binding of the KK10 epitope to the HLA-B27 molecule and impairs infectivity (21–23, 25). The compensatory CA mutation S173A confers WT-level, or more, infectivity to the RK and RKLM mutant viruses (21, 27). Thus, to understand the mechanism, we measured the infectivity of the WT and the RK mutants using replication-competent virus particles. Since, the LM mutation alone does not significantly affect infectivity, this mutant was not included. The titer of the viruses was determined using a qRT-PCR-based lentivirus titration kit (Applied Biological Materials, USA) that estimates virus concentration as infectious units mL^–1^ based on genomic RNA content in viral particles. We inoculated TZM-bl cells (Fig. 3A and B) and Jurkat T-cells (Fig. 3C and D) with equivalent multiplicity of infection (MOI) of WT or the mutant viruses and assessed infectivity 48 hours postinfection (hpi). Luciferase activity in the lysates of TZM-bl cells and intracellular HIV-1 Gag and p24 protein in the Jurkat cells were measured for infectivity. Compared to the WT virus, the infectivity of the RK and the RKLM mutant viruses were drastically reduced. Importantly, the addition of the SA mutation to the RKLM virus restored infectivity to the WT level. These assays using native envelope-containing HIV-1 particles establish that the CTL-escape CA mutation RK severely impairs infectivity and this defect is rescued by the compensatory SA mutation.

### RK-associated infectivity defect is not due to block in the late stages of HIV-1 replication

CA–CA interaction is essential for Gag lattice formation and assembly of immature viruses (62, 63). Cleavage of CA from the Gag precursor is also critical for the formation of the conical capsid of the mature virions (64). Therefore, we probed whether the RK mutations affected Gag processing and particle production. HEK293T cells were transfected with the molecular clones of the WT or the CA mutants and cultured for 48 hours, after which the viral proteins in the cells and the released particles were probed by western blotting (Fig. 3E and F). The Gag processing efficiency in the producer cell was not significantly altered by any of the CA mutations. Further, the CA mutations did not reduce virus release efficiency—measured as the ratio of the amount of virus-associated CA to the total Gag (total amount of virus-associated CA, cell-associated Pr55Gag, cell-associated p41Gag, and cell-associated CA; Fig. 3F). Interestingly, the RKLMSA mutations resulted in a marginal increase in virus release efficiency. These results indicate that the RK mutations do not alter the late stages of HIV-1 replication and suggest a potential postentry block.

### RK mutation minimally affects HIV-1 reverse transcription and nuclear entry

It is evident that the RK and RKLM mutations are not positioned to alter HIV-1 capsid structure (Figs 1 and 2). However, there is indication that these mutations affect reverse transcription of pseudotyped HIV-1 (21). Therefore, we quantified reverse transcription levels in T-cells inoculated with native envelope containing WT and RK mutant viruses. Copy numbers of the late reverse transcription products, in Jurkat or SupT1 cells spinoculated with equivalent MOI of the WT or the mutant viruses, was measured by qPCR. Our results show that the RK and the RKLM mutations did not impose significant alterations in the reverse transcription levels (Fig. 3G and H). The addition of the compensatory SA mutation to the RKLM virus also had no significant effect on reverse transcription. These results diverge from the published work (21) and suggest that the RK mutation-associated infectivity defect arises from a block(s) after the reverse transcription step.

Nuclear import is the next step in HIV-1 replication and prior reports did not show the effect of RK mutation on viral nuclear import (21, 27). So, we measured the nuclear import of the WT and mutant viruses by quantitating the viral 2-LTR circles in infected cells (65), that are commonly used as surrogates of HIV-1 nuclear import (66). The RK and the RKLM mutations did not significantly reduce the levels of nuclear import that could be correlated with the magnitude of the infectivity defect (Fig. 3I and J). Further, the RKLMSA virus did not exhibit any significant difference in the nuclear import levels. As the 2-LTR circles are derivatives of the late reverse transcription products and the RKLM virus showed a marginal reduction in reverse transcription (Fig. 3G and H) and nuclear entry (Fig. 3I and J), we calculated the nuclear import efficiency through the ratio of 2-LTR circles to the reverse transcription (Fig. 3K). No significant difference in the nuclear import efficiency of the WT and mutants was observed, indicating that the RK mutations minimally affect the efficiency of HIV-1 reverse transcription and nuclear import.

### RK mutation blocks proviral integration and SA compensatory mutation restores it

**Fig. 4.**
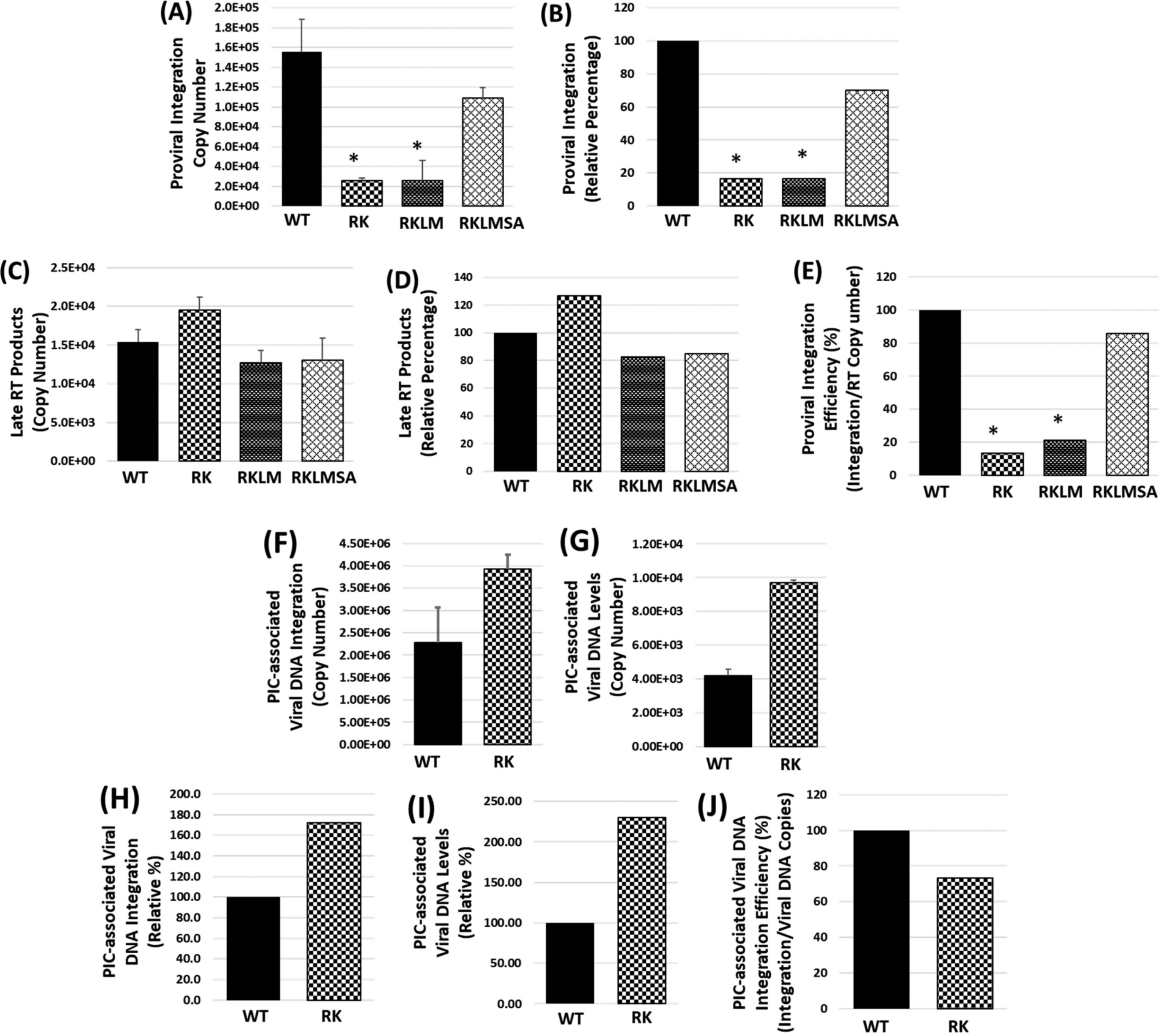
**(A)**–**(E)** Effect of CTL escape RK mutations on HIV-1 integration. **(A)** Quantification of chromosome-integrated viral DNAs and late reverse transcription products in SupT1 cells spinoculated with the WT or mutant viruses for 2 hours at 25°C and cultured for 24 hours at 37°C. Copy number of chromosome-integrated viral DNAs was measured by *Alu*-Gag nested PCR. **(B)** Percentage copy number of chromosome-integrated viral DNA plotted relative to WT. **(C)** Copy number of late reverse transcription products of WT or the mutant viruses as measured by qPCR. **(D)** Percentage late RT products plotted relative to WT. **(E)** Calculated ratio of the copy number of integrated viral DNAs to the respective late reverse transcription products plotted as percentage relative to WT. **(F)**–**(J)** Effect of RK mutation on the integration activity of cytoplasmic PICs in vitro. Integration activity and the viral DNA content of the WT and RK cytoplasmic PICs isolated from Jurkat cells spinoculated with the respective viruses for 2 hours at 25°C and cultured for 5 hours at 37°C were determined. **(F)** Integration activity of the PICs, i.e. the copy number of viral DNAs that were integrated into the target DNA during the in vitro integration assay, as measured by qPCR. **(G)** Copy number of viral DNA content in the cytoplasmic PICs as measured by qPCR. **(H)** Percentage integration activity plotted relative to WT. **(I)** Percentage viral DNA content plotted relative to WT cytoplasmic PICs. **(J)** Ratio of the copy number of integrated viral DNAs to the corresponding PIC-associated viral DNAs plotted as percentage relative to WT. Data shown are representative of at least three independent experiments, with error bars representing the SEMs. * represents *P* < 0.05.

To probe the postnuclear entry block associated with the RK mutation, we compared the levels of chromosomally integrated viral DNA in cells inoculated with WT or the mutant viruses. We used the *Alu*-gag nested PCR assay (67–69), that selectively amplifies the integrated viral DNAs in the 1st round followed by their quantitation in the 2nd round qPCR (70). Jurkat or SupT1 cells spinoculated with equivalent MOI of the WT or the mutant viruses were cultured for 24 hours, and total DNA was isolated from these cells. The copy numbers of proviral DNA were calculated by interpolation from a standard curve generated during the 2nd round qPCR (Fig. 4A and B). As the late reverse transcription products constitute the sole source of the proviral DNAs, the late reverse transcription products in the same samples were also measured by qPCR (Fig. 4C and D). The integration efficiency was calculated by the ratio of the copy numbers of proviral DNAs to the reverse transcription products (Fig. 4E). Strikingly, the results revealed a significant reduction in the proviral DNA levels (Fig. 4A and B) and integration efficiency (Fig. 4E) of the RK and RKLM viruses when compared to the WT infection. Importantly, the addition of the SA mutation to the RKLM virus led to restoration of its integration efficiency (Fig. 4E), which largely correlated with the magnitude of the recovery of the infectivity (Fig. 3A–D). These findings, together with our data of reverse transcription and nuclear import, reveal that the infectivity defect of the RK mutant viruses are primarily due to the dramatic reduction in viral DNA integration.

HIV-1 DNA integration is carried out by the preintegration complex (PIC) (71). Therefore, the integration defect of the RK variant could be the consequence of reduced PIC-associated viral DNA integration activity. To test this, we compared the integration activity of PICs extracted from cells inoculated with the WT or RK virus in vitro. Due to the well-recognized technical challenges with nuclear PIC isolation, we used the cytosolic PIC extracts from Jurkat or SupT1 cells (33, 72, 73). The integration activity of these PICs quantified via a nested PCR assay is the measure of the number of viral DNA copies integrated into a heterologous target DNA. Intriguingly, the integration activity of the RK mutant PICs was higher than that of the WT PICs (Fig. 4F and H; Figure S3A and B, Supplementary Material), in contrast to the significant reduction in the RK mutant proviral DNA integration levels relative to the WT virus (Fig. 4A and B). Furthermore, the viral DNA content of the RK mutant PICs was also higher relative to that of the WT PICs (Fig. 4G and I; Figure S3C and D, Supplementary Material). Notably, the integration efficiency between the WT and the RK mutant PICs did not differ significantly (Fig 4J; and Figure S3E, Supplementary Material). Collectively, these results suggest that the integration activity of the RK mutant PICs is not defective and the RK mutation-associated reduction in proviral integration is not due to faulty assembly or reduced number of PICs.

### CypA depletion restores the integration of the RK mutant virus

Interaction between HIV-1 CA and CypA is essential for viral infection and perturbation of this interaction affects reverse transcription and nuclear import (45, 46, 48, 49, 74, 75). Interestingly, absence of CypA has been reported to rescue the infectivity defect of the RK mutant (21, 26). Because our results indicated that the RK mutation impairs HIV-1 integration, we hypothesized that the rescue of the infectivity defect in CypA-depleted cells is a manifestation of restored integration. To test this, we assessed reverse transcription, nuclear import, and integration of the WT and the RK mutant in the parental (Jurkat _CypA+/+_) and CypA knockout (Jurkat_CypA-/-_) cells (76). These cells were spinoculated with equivalent MOI of the WT or RK mutant virus and the copy numbers of late reverse transcription products, 2-LTR circles, and the proviral DNA were measured by qPCR.

**Fig. 5.**
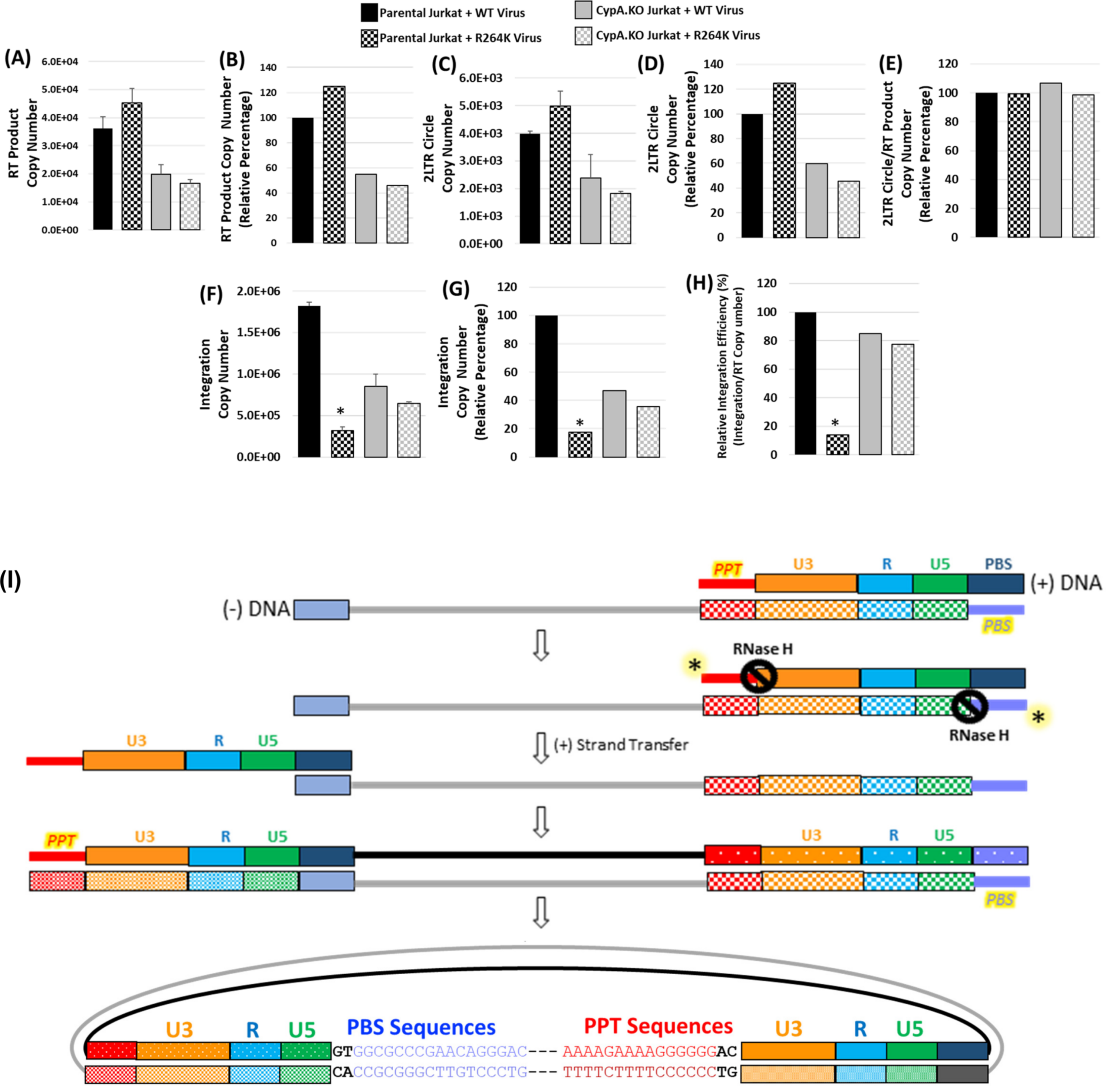
**(A)**–**(H)** Assessment of the effect of RK mutation on viral DNA integration in cells lacking CypA. Copy number of the reverse transcription products, 2-LTR circles, and the chromosome-integrated viral DNAs in the total DNA isolated from the parental or CypA.KO Jurkat cells spinoculated with WT or the RK virus for 2 hours at 25°C and cultured for 24 hours at 37°C were determined. **(A)** Copy number of the late reverse transcription products in the parental or the CypA.KO cells as measured by qPCR. **(B)** Percentage late reverse transcription products plotted relative to WT virus in parental Jurkat cells. **(C)** Copy number of the 2-LTR circles as measured by qPCR. **(D)** Percentage 2-LTR circles plotted relative to WT virus in parental Jurkat cells. **(E)** Ratio of the copy number of 2-LTR circles to the respective late reverse transcription products plotted as percentage relative to WT virus in parental Jurkat cells. **(F)** Copy number of chromosome-integrated viral DNAs as measured by *Alu*-Gag nested PCR. **(G)** Percentage copy number of chromosome-integrated viral DNA plotted relative to WT virus in parental Jurkat cells. **(H)** Ratio of the copy number of chromosome-integrated viral DNAs to the respective late reverse transcription products was calculated and the data plotted as percentage relative to WT virus in parental Jurkat cells. Data shown are representative of three independent experiments, with error bars representing the SEMs. * represents *P* < 0.05. **(I)** Model for the presence of PPT and PBS sequences at HIV-1 DNA ends. Shown schematically is HIV-1 reverse transcription after the completion of the plus-strand strong stop DNA synthesis. In the wild type HIV-1, the RNase H removes the PPT/plus-strand RNA primer (red bold line marked by an asterisk) and the tRNA/minus-strand RNA primer (orchid bold line marked by an asterisk). These RNase H-mediated removal of the PPT and tRNA primers, are essential for the formation of proper viral DNA ends necessary for the integration reaction. However, in the case of the RK mutant HIV-1, failure of RNase H to properly remove the PPT and tRNA primers can lead to the increased retention of these primers at the respective viral DNA ends. These viral DNA ends are unsuitable for the integration in the nucleus and, therefore, are ligated by host enzymes to form 2-LTR circles.

As expected, the RK mutation did not alter the levels of reverse transcription (Fig. 5A and B) and 2-LTR circles (Fig. 5C and D), or the nuclear import efficiency (Fig. 5E) in the Jurkat _CypA+/+_ cells. These data are consistent with the results of Fig. 3(G)–(K), corroborating a block after the nuclear entry step of HIV-1 infection. The ∼2-fold reduction in the reverse transcription of the WT virus in the Jurkat_CypA-/-_ cells illustrate the requirement of CypA during early steps of infection (46). Notably, a comparable ∼2-fold reduction in reverse transcription of the RK mutant in the Jurkat_CypA-/-_ cells also confirm that the infectivity defect of this mutant is not due to a reverse transcription block. Interestingly, assessment of the proviral DNA copies and the the integration efficiency (calculated by normalizing the proviral DNA levels to the corresponding late reverse transcription products), as well as plotting of the data as percentage relative to WT virus in parental Jurkat cells (Fig. 5F–H), revealed that the integration of the RK mutant virus did not differ significantly from that of the WT virus in CypA-depleted cells. Collectively, these findings suggest that the rescue of the infectivity defect of the RK mutant in CypA-depleted cells is primarily due to the restoration of the viral DNA integration.

### RK mutant viral DNA ends are appended with aberrant viral sequences

Our data showing that the RK mutation significantly inhibits integration without affecting reverse transcription, nuclear import, and PIC function was intriguing. We hypothesized that the reduction in integration becomes evident only after the PIC enters the nucleus. Therefore, we tested whether the RK mutation compromises the integrity of the viral DNA ends that is necessary for chromosomal integration. The 2-LTR circles, which are generated in the nucleus (65, 77) represent an elegant tool to probe the integrity of the viral DNA ends (78–80). Thus, we examined the nucleotide sequences of the 2-LTR circle junctions in cells inoculated with the WT or the RK mutant virus. The junctions of the 2-LTR circles from infections of each virus were first amplified by nested PCR, cloned into a plasmid vector, and the sequences of the junctions were determined by Sanger sequencing and analyzed by multiple alignment.

Results from this analysis revealed two interesting patterns (Table 1; Figure S4A and B, Supplementary Material). First, compared to the WT virus, a higher proportion of the RK mutant viral DNA ends were appended with long aberrant viral sequences. Second, a significantly higher proportion of these aberrant sequences mapped to the viral poly-purine tract (PPT) and/or the tRNA/primer-binding site (PBS) sequences. Remarkably, the proportion of the compensatory RKLMSA mutant viral DNAs with aberrant PPT and/or PBS sequences were minimal (Table 1; Figure S4C, Supplementary Material). Further studies are warranted to ascertain whether these alterations directly contribute to the impaired integration of the HIV-1 CTL escape mutants.

**Table 1.** Assessment of the effect of KK10-linked CTL escape mutations on the integrity of the termini of the reverse transcription products (viral DNAs).

		WT	RK	RKLMSA
Total number of sequences analyzed		104	106	166
2-LTR junctions without aberrant nucleotide insertions	Complete 3’-end processing of both LTR ends, with intact flanking regions	0	0	0
	Complete 3’-end processing of both LTR ends, accompanied by deletions in flanking regions	6	14	15
	Complete 3’-end processing of one LTR end (5’U3 or 3’U5)	1	7	14
	No 3’-end processing of both LTR ends	15	7	13
	Partial 3’-end processing of one LTR end	20	12	10
2-LTR junctions with aberrant nucleotide insertions	Complete 3’-end processing of both LTR ends, with intact flanking regions	0	1	1
	Complete 3’-end processing of both LTR ends, accompanied by deletions in flanking regions	2	1	8
	Complete 3’-end processing of one LTR end (5’U3 or 3’U5)	21	32	34
	No 3’-end processing of both LTR ends	35	26	59
	Partial 3’-end processing of one LTR end	5	6	22
Source of aberrant insertion sequences	Number of clones with insertions at the junction (% of total sequences)	63 (60.6%)	66 (62.3%)	119 (71.7%)
	Insertions mapping to PPT sequences (% of total insertion sequences)	4 (6.3%)	16 (24.2%)	7 (5.9%)
	Insertions mapping to PBS sequences (% of total insertion sequences)	8 (12.7%)	25 (37.9%)	9 (7.6%)

Junctions of the 2-LTR circles present in the total DNA isolated from Jurkat cells spinoculated with the WT or mutant viruses for 2 hours at 25°C and cultured for 24 hours at 37°C were amplified by a nested PCR strategy, cloned into a plasmid vector, and the DNA sequences determined by Sanger sequencing were aligned and analyzed. The total number of 2-LTR junction sequences analyzed for the WT and the mutant viruses is indicated at the top of the table. The total number of each type of 2-LTR junction sequence identified in the analyses is shown for the WT, RK, and RKLMSA virus-inoculated samples. Shown at the bottom of the table are the total number of 2-LTR junctions containing aberrant insertion sequences and their percentage relative to the total sequences analyzed, and the total number of insertion sequences mapping to the PPT or PBS and their percentage relative to the total insertion sequences. See also Figure S4 (Supplementary Material).

## Discussion

The CTL response against the HIV-1 KK10 epitope has been linked to robust virus control and delayed disease progression in HLA-B27-positive infected individuals. Selection of the CA mutation RK, associated with viral escape, disrupts epitope binding to HLA-B27 but also causes a severe infectivity defect. The compensatory CA mutation SA relieves the RK-associated infectivity defect and triggers loss of viremia control and progression to AIDS. In this study, we have identified impaired HIV-1 integration as the principal mechanism underlying the infectivity defect of the RK mutant virus and the SA compensatory mutation largely rescues the integration defect of the CTL-escape RK mutant virus.

Specific mutations in the genetically fragile CA compromise capsid integrity and disrupt early events of HIV-1 replication. While Schneidewind et al. (21) reported that the RK mutation had no significant effect on capsid stability, Schommers et al. (27) suggested a modest increase in the stability of the RK mutant capsid. Notably, the RK mutation does not appear to overlap with the critical structural elements of the capsid. Our molecular modeling and MD simulations demonstrated that the RK mutation neither altered the integrity of the hexameric, pentameric, or the tubular structure of the capsid (Figs 1 and 2). Further, the RK mutation minimally affected interaction with CypA- the host factor that modulates HIV-1 capsid stability.

Our results confirmed that the RK mutation—independently or in combination with the LM mutation—causes loss of infectivity that is rescued by the compensatory SA mutation (Fig. 3). Interestingly, our infection assays carried out in an epithelial cell line and in a T-cell line with native envelope-containing viruses inform that the consequence of the RK, RKLM, and RKLMSA mutation on infectivity are not dependent on cell types. Further, the infectivity defect of these variants irrespective of the presence of the native HIV-1 envelope or the VSVg-pseudotyped envelope (21, 27) suggests that the mode of viral entry does not influence how these CA mutations impair infectivity. Our results of the Gag processing and virus release efficiency also preclude effect of these mutations on late stages of infection. Therefore, the RK-associated infectivity defect most likely arises from impairment of the postentry step(s) of infection.

Our findings that the RK mutations minimally perturb capsid integrity and its interaction with CypA suggested that these CA mutations may not affect the reverse transcription efficiency. However, Schneidewind et al (21) reported that the RK mutation, despite having no effect on the capsid stability, caused a severe reduction in reverse transcription. In contrast, our data did not show significant difference in reverse transcription between the WT and the RK mutant viruses (Fig. 3G and H). While Schneidewind et al. (21) used VSVg-pseudotyped virus that enters target cells by endocytosis, we used native envelope-containing viruses that enters by CD4-receptor-mediated membrane fusion. This raises the possibility of a pseudotyping-associated effect on the RK mutant reverse transcription, since VSVg pseudotyping can alter the phenotype of certain CA mutants (81, 82).

The lack of a significant effect of the RK mutation on reverse transcription implies that this CA mutation disrupts the downstream step(s) in HIV-1 infection. Our quantification of the 2-LTR circles showed no significant differences in the levels of nuclear import of the WT or RK mutant viruses (Fig. 3 I–K). It is increasingly becoming clear that the interaction between the HIV-1 CA and the host CPSF6 is essential for optimal nuclear import of the virus. Interestingly, results from our molecular modeling, MD simulations, and Y2H assays (Fig. 2) showing no significant effect of the RK mutations on the interaction between CA and CPSF6 further supports our 2-LTR circle data and the improbability of a RK mutation-induced defect in viral nuclear import. Strikingly, measurement of the proviral copy numbers revealed that the CTL escape CA mutations RK and RKLM are severely impaired to integrate into the host chromatin (Fig. 4A and B). Accordingly, the compensatory SA mutation significantly rescued the viral DNA integration defect of the RK and RKLM mutant viruses. Taken together, these results provide strong evidence that the RK mutation-associated reduction in infectivity is not due to alterations in reverse transcription and nuclear import but exclusively a consequence of impaired viral DNA integration.

It is important to note that a lack of a concomitant increase in the levels of 2-LTR circles despite a defect in HIV integration is not without precedence. For instance, while knockdown of TNPO3 caused a significant reduction in integrated viral DNA levels, the levels of late RT products and 2-LTR circles were comparable between control and TNPO3 knockdown cells (83). Importantly, these authors reported that the levels of 2-LTR circles remained unchanged in TNPO3-depleted cells even upon treatment with the integrase inhibitor Raltegravir. Therefore, the comparable levels of 2-LTR circles in WT and RK mutant virus-infected cells could suggest an impairment in the formation or stability of 2-LTR circles in the nucleus due to RK mutation..

Integration of the viral DNA into the host chromosome is carried out by the HIV-1 PIC. Therefore, a diminished integration activity of the RK PICs could be the underlying cause of impaired viral DNA integration. Our results showed that the viral DNA levels in the isolated WT and RK mutant cytoplasmic PICs were comparable, thereby further corroborating that the RK mutation has no significant effect on reverse transcription. Unexpectedly, the integration activity of the WT and RK mutant PICs did not vary significantly (Fig. 4F and H), in striking contrast to a significant reduction in proviral integration (Fig. 4A and B). There are key differences in our in vitro and the proviral integration assays. These include contrasting nature of the target DNAs (naked DNA vs chromosomal DNA), distinct composition of the cytoplasmic PICs and nuclear PICs (e.g. reduction in PIC-associated integrase and CA levels), and spatial and temporal constraints in the involvement of cellular cofactors (e.g. nuclear CPSF6 and LEDGF). Although these differences may play a role, it should be noted that our measurements of reverse transcription are quantitative but not qualitative. Because proviral integration is dependent on precise viral DNA ends, we investigated whether the quality of the viral DNAs in the nucleus are incompetent for integration.

A key step leading up to HIV-1 integration is the 3’-end processing of viral DNAs containing precisely defined termini. The generation of viral DNA with canonical termini depends on completion of strand transfer reactions and the proper removal of the tRNA and the PPT primers used for the minus strand and plus strand DNA synthesis, respectively. Any disruption of the above events results in viral DNA termini that are incompetent for integration and, consequently, are circularized in the nucleus by host machinery into 2-LTR circles. Therefore, the sequence of the junctions of 2-LTR circles can be used to assess the integration competency. Accordingly, the 2-LTR circle junction sequences are typified by the presence of unprocessed viral DNA ends, deletions within the 3’U5 or 5’U3 region, and insertion (i.e. retention) of aberrant viral DNA mapping mostly to PPT and PBS sequences. Indeed, we observed all these modifications in our comparative analysis of the 2-LTR circles from cells inoculated with the WT or RK mutant virus (Table 1). However, compared to the WT, the insertions in RK 2-LTR circle junctions presented two distinctive patterns. (i) A higher proportion of these insertions were significantly longer. (ii) A significantly higher proportion of these insertions mapped to the viral PPT and/or the PBS sequences. This atypical and enhanced retention of the PPT and PBS sequences at the RK mutant viral DNA termini could be a major factor contributing to its impaired integration. Remarkably, compared to the RK mutant viral DNAs, the proportion of the RKLMSA mutant viral DNAs appended with aberrant PPT and/or PBS sequences were significantly low. It is well-established that proper and precise functioning of the RNase H is critical for the generation of viral DNA with defined ends (79, 80). Overall, it has been suggested that the presence of aberrant PPT-derived sequences could arise from a decrease in RNase H activity or altered RNase H specificity or a combination of both (79, 80). However, it is unclear how the RK mutation that is positioned in the CA and did not cause any significant alterations in the reverse transcription levels can selectively alter the RNase H activity. Further, autointegration is unlikely to be the source of these viral DNA insertions for several reasons. (i) Primers used for obtaining the 2-LTR circle junction sequences are specific to 2-LTR sequences. (ii) Our use of 24-hpi sample excludes the detection of autointegrants because they accumulate at early time points (∼10 hpi) (84) and are rapidly depleted by 24 hpi. (iii) Requirement of viral DNA 3’-end processing for autointegration is incompatible with our sequencing data showing that majority of the viral DNA termini are unprocessed or partially processed. Nevertheless, we speculate that the retention of the PPT and PBS sequences at RK mutant viral DNA ends may significantly contribute to the impaired integration. Furthermore, whether these alterations in the viral DNA ends are due to a qualitative glitch in reverse transcription requires further study.

The rescue of the infectivity defect of the RK mutant in CypA-depleted cells and in cells treated with cyclosporin A indicates that an interaction between the RK mutant CA and CypA is a prerequisite for the infectivity defect (21, 27). Is CypA playing a causative role in the RK mutant infectivity defect? Interestingly, supportive evidence comes from our data comparing the viral DNA integration levels of the RK mutant with that of the WT virus in the CypA-depleted cells (Fig. 5). In line with the infectivity assays, the integration level of the RK mutant was comparable to that of the WT virus in the CypA knockout cells. Especially striking is the several fold increase in integration of the RK mutant virus in the CypA knockout cells relative to the control Jurkat cells. This indicates that CypA and its binding to the mutant CA is a prerequisite for the significant reduction in integration of the RK mutant. Does this also imply that the reduction in infectivity by RK mutation is exclusively a consequence of the CypA-associated impaired viral DNA integration? In agreement with the findings from the control Jurkat cells, the reverse transcription and nuclear import levels of the RK mutant was comparable to that of the WT virus in the CypA-depleted cells. This indicates that the CypA depletion-mediated restoration of the RK mutant infectivity is primarily an outcome of the rescue of the RK mutation-induced integration defect. It should be noted that the prior studies did not assess the effect of these CA mutations on HIV-1 integration (21, 27) and, instead, the reduction in infectivity of the RK and mutant viruses in single-cycle infectivity assays were attributed to the reduction in reverse transcription levels (21).

Finally, our study raises a number of broadly important questions, including: (1) How does the RK mutation leads to the generation of imperfect viral DNA ends? (2) Why is CypA required for the RK mutation-associated integration defect? and (3) How does the compensatory CA mutation SA restore the RK mutant infectivity without disrupting the interaction between the mutant CA and CypA? The RK mutation appears to neither disrupt the interaction between CA and CypA nor alter the quantitative aspects of reverse transcription. However, it is possible that the interaction between the RK mutant CA and CypA leads to an atypical and prolonged association between these proteins. This, in turn, could alter the qualitative aspects of reverse transcription (e.g. RNase H activity) and generate viral DNA containing integration-incompetent termini. Conversely, the compensatory SA mutation or absence of CypA may preclude such association between the RK mutant CA and CypA. However, the ability of the SA mutation to restore the RK virus integration without imposing any loss of interaction between the mutant CA and CypA, suggest possible involvement of additional mechanisms. Interestingly, escape from CypA-dependent restriction has been also reported in the case of the CTL-escape CA mutant virus selected in response to the HLA-B57 targeting of the CA-derived TW10 epitope (85). While selection of the T242N CA mutation in the TW10 epitope helps HIV-1 escape from the CTL response, the accompanying reduction in infectivity is rescued only upon selection of the compensatory CA mutations in the CypA-BL that potentially disrupt or alter CA binding to CypA. It is noteworthy that the CTL-escape CA mutant viruses R264K and T242N, selected in response to targeting of different viral peptide epitopes by HLA-B27 and HLA-B57, respectively, are equally subject to the CypA-dependent restriction activity. Accordingly, CPSF6 was reported as a requirement for the infectivity defect of the RK mutant (26). Although there is no evidence linking the interaction between CA and CPSF6 to reverse transcription, a recent study reported that CypA binding to HIV-1 capsid prevents cytoplasmic CPSF6 from binding and disrupting the capsid (86, 87). An alternative possibility, from our data showing that the cytoplasmic PICs are competent for integration, is that the RK mutation-associated aberrant viral DNA termini are generated only in the nucleus. For instance, an atypical association between RK mutant CA and CypA may delay viral integration during which the viral DNA termini may be subjected to host DNA polymerase-mediated copying and insertion, resulting in the generation of aberrant viral DNA termini.

## Materials and Methods

### Cell culture and proviral plasmids

The HEK293T (ATCC) and TZM-bl (John C. Kappes, Xiaoyun Wu, and Tranzyme, Inc) cell lines were cultured in DMEM (Gibco) supplemented with 10% heat-inactivated FBS (Gibco), 2  mM glutamine, 100  U mL^–1^ penicillin, and 100  μg mL^–1^ streptomycin. The SupT1 (ATCC) and the Jurkat E6-1 and Jurkat CypA-/- (Douglas Braaten and Jeremy Luban) cell lines were cultured in RPMI 1640 medium supplemented with 10% heat-inactivated FBS, 2  mM glutamine, 100  U mL^–1^ penicillin, and 100  μg mL^–1^ streptomycin. Cells were cultured at 37°C with 5% CO_2_. Viruses were generated from the full-length HIV-1 molecular clone pNL43 and its mutant derivatives. Details about the construction of the pNL43 molecular clones harboring CA mutations are included in the Supplementary Materials and Methods.

### Molecular modeling studies

The initial coordinates of the HIV-1 CA hexamer were generated by applying a 6-fold symmetry operation onto a native full-length HIV-1 capsid protein (PDB accession code 4XFX). The two loops between residues 5 to 9 and residues 222 to 231, missing in the original structure, were built by Modeler (88). Three models of CA hexamer mutations, R132K (RK), R132KL136M (RKLM), and R132KL136MS41A (RKLMSA), were generated in VMD (89) Mutator Plugin from the 4XFX hexamer model. After placed an IP6 molecule (55) close to the Arg18 ring, sodium and chloride ions were introduced around these models, based on the local electrostatic potential, using CIONIZE plugin in VMD. Subsequently, all models were then solvated with CHARMM TIP3P water model (90) and the total NaCl concentrations were set to 150 mM, resulting charge-neutral systems of about 65 K atoms.

Details about the molecular modeling of two CA pentamer structures were described in Xu et al. (56). Briefly, the coordinates of the disulfide stabilized pentamer were from crystal structure 3P05, after mutating the cysteine residues back to the HIV-1NL4-3 wildtype sequence. The coordinates of the missing residues in the PDB model were built by Modeler (88). Using the mutated 3P05 pentamer structure for the initial coordinates, the 5MCY pentamer model was generated by running molecular dynamics flexible fitting (MDFF) of the initial coordinates into 5MCY cryo-EM density (EMBD: EMD-3466).

Simulation protocol: The solvated systems were then subjected to minimization in two stages, both using the conjugated gradient algorithm (91) with line search (92). Each stage consisted of 10,000 steps of energy minimization. During the first stage, only water molecules and ions were free to move, while the protein and IP6 molecule were fixed. In the second stage, the backbone atoms of the CA protein were applied with a harmonic restraint with a force constant of 10.0 kcal mol^–1^ Å^–2^. Convergence of the minimization procedure was confirmed once the variance of the gradient was below 0.1 kcal mol^–1^ Å^–1^. Following minimization, the systems were tempered from 50 to 310 K in increments of 20 K over 1 ns. Subsequently, the systems were equilibrated at 310 K for 100,000 steps, while the protein backbone atoms were restrained. Then positional restraints were gradually released at a rate of 1.0 Kcal mol^–1^ Å^–2^ per 400 ps from 10.0 Kcal mol^–1^ Å^–2^ to 0.0 Kcal mol^–1^ Å^–2^.

All MD simulations in present study were performed with NAMD2.13 (93) using CHARMM force fields (94, 95). In present study, an internal time step of 2 fs was employed in the multistep vRESPA integrator as implemented in NAMD, bonded interactions were evaluated every 2 fs. Temperature was held constant at 310 K using a Langevin thermostat with a coupling constant of 0.1 ps^–1^. Pressure was controlled at 1 bar using a Nose–Hoover Langevin piston barostat with period and decay of 40 ps and 10 ps, respectively. The Shake algorithm was employed to constraint vibrations of all hydrogen atoms. Long range electrostatics was calculated using the particle-mesh-Ewald summation with a grid size of 1 Å and a cutoff for short-range electrostatics interactions of 12 Å. MD result analysis: The RMSD, RMSF, contact and ion occupancy analyses were performed in VMD (89). The APBS software (96) was employed to compute the electrostatic potential surfaces. The sodium and chloride ions were added to all systems according to the local coulombic potential by CIONIZE in VMD (89).

### GAL4-based Y2H assay

Details of the construction of the Y2H plasmids and evaluation of protein interactions by the GAL4-based Y2H assay are described in detail in the Supplementary Materials and Methods.

### Cell- and virus-associated viral protein expression

HEK293T cells were transfected with WT or mutant proviral plasmid DNAs and, 24 or 48 hours post-transfection, the culture media containing the released virus particles (virus fraction) was collected, and the cells were lysed in TX-100 lysis buffer (300 mM NaCl; 50 mM Tris-HCl, pH 7.5; 0.5% Triton X-100; β-mercaptoethanol, Sigma protease inhibitor cocktail) for 10 min on ice. The virus pellet, obtained by centrifugation of virus fraction at 32,000 rpm for 45 min at 4°C, was lysed in 0.1 mL of TX-100 lysis buffer for 10 min on ice. Cell lysates and virus lysates were resolved by SDS-PAGE, transferred onto nitrocellulose membrane, and probed with mouse anti-CA monoclonal antibody (1:1,000 dilution; 183-H12-5C; NIH AIDS Reagent Program) followed by secondary HRP-conjugated goat antimouse IgG(H + L) (1:10,000 dilution; Bio-Rad). Virus release efficiency is calculated as the amount of virus-associated CA divided by the total Gag (virus-associated CA + cell-associated Pr55Gag + cell-associated p41Gag + cell-associated CA).

### Preparation of virus stocks, determination of titer, and infectivity assays

For infectivity assays, TZM-bl cells, were cultured overnight, were inoculated with WT or mutant viruses (MOI of 0.05 or 1.0) in the presence of polybrene for 2 hours at 37°C/5% CO_2_. 48 hpi, the cells were washed with and the luminescence activity in the cell lysates was measured using the Luciferase Assay. For infection of Jurkat T cells, the cells were spinoculated with virus stocks (MOI of 1.0) and were cultured for 24 or 48 hours at 37°C. The cells were collected, pelleted, and after a wash with PBS, were processed for FACS analysis. The details of these assays are described in the Supplementary Materials and Methods

### HIV-1 infection of target cells for quantitative assays by qPCR

Jurkat or SupT1 cells were spinoculated with virus stocks for 2 hours at 25°C and then were cultured for 24  hours at 37°C. The total DNA isolated from these cells were used in quantitative assays. A SYBR green-based qPCR was used to quantify the reverse transcription products, and a TaqMan probe-based qPCR was used to quantify the 2-LTR circles. The copy number of (chromosomally integrated) proviral DNA in HIV-1-inoculated cells was determined using the *Alu*-gag nested PCR as described previously (33). Details of these qPCR based quantifications are included in the Supplementary Materials and Methods.

### Isolation of HIV-1 cytoplasmic PICs and measurement of integration activity in vitro

The target cells (10  ×  10^6^) were spinoculated with virus stocks at 480  ×  *g* for 2  hours at 25°C. The spinoculated cells were collected and pelleted by centrifugation, and after removing the supernatants, were resuspended in new RPMI complete medium and cultured for 5  hours at 37°C. The HIV-1 PICs were then isolated from the cell samples using a published protocol (33) with modifications that included two washes with 1  mL of buffer K^−/−^ followed by cell resuspension in 0.5  mL of ice-cold buffer K^+/+^. In vitro integration assays of cytoplasmic PICs were performed as described previously (33) and the details are included in the Supplementary Materials and Methods.

### Sequence analysis of junctions of 2-LTR circles

A nested PCR strategy was used to amplify the junction of the 2-LTR circles in HIV-1-infected cells. The resulting PCR amplicon was gel-purified and ligated to pGEMT-Easy vector (Promega), as per the manufacturer-recommended protocol. The sequence of the 2-LTR junction DNA inserts in the recombinant plasmids was determined by Sanger DNA sequencing using primers flanking the cloning site, and multiple alignment was used to analyze the sequence data. Details are included in the Supplementary Materials and Methods.

### Statistical analyses

Data were expressed as mean ± SEM obtained from three independent experiments. Significance of differences between control and treated samples was determined by Student's t test. Values of *P* < 0.05 were considered to be statistically significant.

## Supplementary Material

pgac064_Supplemental_File

## Data Availability

The authors declare that all data is included in the manuscript and/or supporting information.
