# Supplementary material for: HIV-1 mutants that escape the cytotoxic T-lymphocytes are defective in viral DNA integration
Source: PNAS Nexus. 2022 May 20;1(2):pgac064. doi: 10.1093/pnasnexus/pgac064 (PMC9198661; doi:10.1093/pnasnexus/pgac064)
Supplement: pgac064_Supplemental_File [file pgac064_supplemental_file.pdf]

Supplementary Information:

HIV-1 Mutants that Escape the Cytotoxic T-Lymphocytes are Defective in Viral DNA Integration

Muthukumar Balasubramaniam[1,2*], Benem-Orom Davids[1,2,3], Alex Bryer[5], Chaoyi Xu[5], Santosh Thapa[1,2], Jiong Shi[6], Christopher Aiken[6], Jui Pandhare[1,3,4], Juan R. Perilla[5], and Chandravanu Dash[1,2*]

[1]The Center for AIDS Health Disparities Research, [2]Department of Biochemistry and Cancer Biology, [3]School of Graduate Studies and Research, [4]Department of Microbiology and Immunology, Meharry Medical College, Nashville, Tennessee.
[5]Department of Chemistry, University of Delaware, Newark, Delaware.
[6]Department of Microbiology, Pathology, and Immunology, Vanderbilt University Medical Center, Nashville, Tennessee.

Correspondence

Muthukumar Balasubramaniam, PhD
Old Hospital Bldg-Room 5001, Meharry Medical College,
1005 Dr. DB Todd Jr. Blvd, Nashville, TN 37208, USA
Email: muthukumarb@mmc.edu
And
Chandravanu Dash, PhD
Old Hospital Bldg-Room 5027, Meharry Medical College,
1005 Dr. DB Todd Jr. Blvd, Nashville, TN 37208, USA
Email:cdash@mmc.edu

This file includes:
SI Materials and Methods
Figures S1 to S4
Legends for Figures S1 to S4

MATERIALS AND METHODS

Proviral plasmids. Viruses were generated from the full-length HIV-1 molecular clone pNL43 and its mutant derivatives. Site-specific mutations in the capsid (CA) coding region were first introduced, using Q5 Site-Directed Mutagenesis Kit (NEB) and custom primers, into a 1310-bp BSSHII/ApaI DNA fragment of pNL43 cloned into the pMiniT 2.0 vector (NEB). R264K: 5'-ATCTATAAAAAATGGATAATCCTG-3' and 5'-TTCTCCTACTGGGATAGG-3'; L268M: 5'-ATGGATAATCATGGGATTAAATAAAATAG-3' and 5'-CTTTTATAGATTTCTCCTACTG-3'; addition of R264K to pMiniT-L268M: 5'-AGGAGAAATCTATAAAAAATGGATAATCATGG-3' and 5'-ACTGGGATAGGTGGATTATGTG-3'; addition of S173A to pMiniT-L268M+R264K: 5'-ACCCATGTTTGCAGCATTATC-3' and 5'-ATTACTTCTGGGCTGAAAG-3'. The BssHII-ApaI DNA fragments with the mutations were individually ligated to the BssHII+ApaI-cut pNL43 plasmid to yield pNL43-RK, pNL43-LM, pNL43-RKLM, and pNL43-RKLMSA plasmids, respectively.

GAL4-based yeast two-hybrid (Y2H) assay. The pNL43 CA ORF was amplified by PCR using primers 5'-TAAGCAGAATTCCCTATAGTGCAGAACCTCCAGG-3' and 5'-TCATTAGTCGACTATCACAAAACTCTTGCTTTATGG-3', and the EcoR1 and Sal1-digested PCR amplicon was ligated to the EcoR1+Sal1-cut pGBKT7 plasmid or EcoR1+Xho1-cut pGADT7 plasmid to yield the pGBKT7-CA and pGADT7-CA (Clontech), respectively. The mCherry ORF was amplified from mCherry-H2A-10 (Addgene plasmid# 55054) by PCR using primers 5'-TGACAGAATTCATGGTGAGCAAGG-3' or 5'-TGACACTCGAGTTACTTGTACAGC-3', and the EcoR1 and Sal1-digested PCR amplicon was ligated to the EcoR1+Sal1-cut pGBKT7 plasmid or EcoR1+Xho1-cut pGADT7 plasmid to yield the pGBKT7-mCherry and pGADT7-mCherry, respectively. The CypA ORF, released from pET21-CypA plasmid by digestion with Nde1 and Xho1 enzymes, was ligated to Nde1+Xho1-cut pGADT7 plasmid to yield the pGADT7-CypA. The R264K/G791A mutation was introduced in the CA ORF in the pGBKT7-CA and pGADT7-CA plasmids using primers: 5'-ATCTATAAAAAATGGATAATCCTG-3' and 5'-

TTCTCCTACTGGGATAGG-3’, to yield the pGBKT7-CA-RK and pGADT7-CA-RK, respectively. The CPSF6 ORF was amplified from pTarget-CPSF6 by PCR using primers 5’-TATAGAATTCATGGCGGACGGCGTGG-3’ and 5’-TATACTCGAGCTAACGATGACGATATTCGCGCTC-3’, and the EcoR1 and Xho1-digested PCR amplicon was ligated to the EcoR1+Xho1-cut pGADT7 plasmid to yield pGADT7-CPSF6. Evaluation of protein interactions by the GAL4-based Y2H system was performed by following the manufacturer’s recommendations for the Matchmaker GAL4 Two-hybrid System 3 (Clontech).

Cell- and virus-associated viral protein expression. HEK293T cells were transfected with WT or mutant proviral plasmid DNAs and, 24 h or 48 h post transfection, the culture media containing the released virus particles (virus fraction) was collected, and the cells were lysed in TX-100 lysis buffer (300mM NaCl; 50mM Tris-HCl, pH 7.5; 0.5% Triton X-100; β-mercaptoethanol, Sigma protease inhibitor cocktail) for 10 min on ice. The virus pellet, obtained by centrifugation of virus fraction at 32,000 rpm for 45 min at 4°C, was lysed in 0.1 mL of TX-100 lysis buffer for 10 min on ice. Cell lysates and virus lysates were resolved by SDS-PAGE, transferred onto nitrocellulose membrane, and probed with mouse anti-CA monoclonal antibody (1:1000 dilution; 183-H12-5C; NIH AIDS Reagent Program) followed by secondary HRP-conjugated goat anti-mouse IgG(H+L) (1:10,000 dilution; Bio-Rad). Virus release efficiency is calculated as the amount of virus-associated CA divided by the total Gag (virus-associated CA + cell-associated Pr55Gag + cell-associated p41Gag + cell-associated CA).

Preparation of virus stocks and determination of titer. Virus stocks were generated by PEI-mediated transfection of HEK293T cells with proviral plasmid constructs. Briefly, 2×10^6 HEK293T cells, seeded per 10-cm culture dish and cultured overnight, were transfected with 10 or 20 μg of plasmid DNA. After 12-16 h, the cells were washed with 1X PBS and cultured in new DMEM complete medium for additional 48 h at 37°C. The virus-containing culture media was harvested,

centrifuged at low-speed, filtered through 0.45-μm filters, and treated with DNase I (Calbiochem/MilliporeSigma; 20 μg/mL of supernatant) in the presence of 10 mM magnesium chloride for 1 h at 37°C. The virus titer was determined by using the qPCR lentivirus titration kit (Applied Biological Materials) as per the manufacturer-recommended protocol.

HIV-1 infectivity assay. TZM-bl cells, seeded at 4×10^4 cells per well in 24-well plate and cultured overnight, were inoculated with WT or mutant viruses (MOI of 0.05 or 1.0) in the presence of polybrene for 2 h at 37°C/5% CO_2. The cells were washed with 1X PBS and cell culture medium and were cultured in new DMEM complete medium for 48 h at 37°C. The cells were washed with 1X PBS, lysed in 1X GLO lysis buffer (Promega) and the luminescence activity in the resulting cell lysates was measured using the Luciferase Assay System (Promega).

Jurkat T cells (2.5×10^5) were spinoculated with virus stocks (MOI of 1.0) at 480 × *g* for 2 h at 25°C. The spinoculated cells were collected and pelleted by centrifugation, and after removing the supernatants, were resuspended in new RPMI complete medium and cultured for 24 h or 48 h at 37°C. The cells were collected, pelleted, and after a wash with PBS, were resuspended in 300 μL of BD Cytofix/Cytoperm (BD Biosciences) and incubated for 20 min at 4°C. The cells were then pelleted, washed with 300 μL of BD Perm/Wash buffer, and resuspended in 110 μL of BD Perm/Wash buffer. Each cell sample (50 μL) was incubated at 4°C for 20 min with 1 μL of KC57-FITC antibody (Beckman Coulter Life Sciences) that identifies the HIV-1 Gag and p24, washed twice with 300 μL of BD Perm/Wash buffer, and resuspended in 100 μL of BD Perm/Wash buffer. The percentage of FITC-labelled cells, as a marker for HIV-1 infectivity, was determined by FACS analysis on Guava easyCyte HT (Millipore Sigma).

HIV-1 infection of target cells for quantitative assays. Jurkat or SupT1 cells (1×10^6 cells or 2×10^6 cells per well) in 24-well plates were spinoculated with virus stocks (MOI of 0.05 or 0.1) at 480 × *g* for 2 h at 25°C. The spinoculated cells were collected and pelleted by centrifugation, and

after removing the supernatants, were resuspended in new RPMI complete medium and cultured for 24 h at 37°C. The cells were collected, pelleted, and after a wash with PBS, resuspended in 150 µL of PBS or nuclease-free water, followed by isolation of total DNA using the Quick-DNA miniprep kit (Zymo Research) according to the manufacturer's instructions.

Quantitation of reverse transcription products and 2-LTR circles by qPCR. A SYBR green-based qPCR was used to quantify the reverse transcription products, and a TaqMan probe-based qPCR was used to quantify the 2-LTR circles. The qPCRs were assembled in triplicates using 100 ng of sample DNA, 1× iTaq Universal SYBR Green Supermix or 1× iTaq Universal Probe Supermix (Bio-Rad), 300 nM each of forward primer (late RT product: MH531/5′-TGTGTGCCCGTCTGTTGTGT-3′; 2-LTR circle/5′-AACTAGGGAACCCACTGCTTAAG-3′) and reverse primer (late RT product: MH532/5′-GAGTCCTGCGTCGAGAGAGC-3′; 2-LTR circle/5′-TCCACAGATCAAGGATATCTTGTC-3′), and, in the case of probe-based qPCR, 100 nM TaqMan probe (5′-[FAM]-ACACTACTTGAAGCACTCAAGGCAAGCTTT-[TAMRA]-3′). The qPCRs were carried out under the following thermocycling conditions: late RT products- 95°C for 3 min, followed by 39 cycles of amplification and acquisition at 94°C for 15 s, 58°C for 30 s, and 72°C for 30 s; 2-LTR circles- 95°C for 3 min, followed by 39 cycles of amplification and acquisition at 94°C for 15 s, 58°C for 30 s, and 72°C for 30 s. The CFX Manager software (Bio-Rad) was used to determine the copy number of the target DNA in the qPCR samples by plotting the data against the respective standard curve that was generated in parallel using 10-fold serial dilutions of known copy numbers (10^0 to 10^8) of pNL43 (for late RT products) or the p2LTR plasmid containing the 2-LTR junction sequence (for 2-LTR circles).

Quantitation of proviral DNAs by nested PCR. The copy number of (chromosomally integrated) proviral DNA in HIV-1-infected cells was determined using the *Alu*-gag nested PCR as described previously[58]. The first-round PCR included 100 ng of sample DNA and the primers 5′-

GCCTCCCAAAGTGCTGGGATTACAG-3 and 5′-GTTCCTGCTATGTCACTTCC-3′. and was carried out at: 95°C for 5 min, followed by 23 cycles of amplification at 94°C for 30 s, 50°C for 30 s, 72°C for 4 min, and a final extension at 72°C for 10 min. The second-round qPCR included one-tenth volume of the first-round PCR products, 1× iTaq Universal Probe Supermix (Bio-Rad), primers 5′-TCTGGCTAACTAGGGAACCCA-3′ and 5′-CTGACTAAAAGGGTCTGAGG-3′, and the TaqMan probe 5′-[6-FAM]-TTAAGCCTCAATAAAGCTTGCCTTGAGTGC-[TAMRA]-3′, and was carried out at: 95°C for 3 min, 39 cycles of amplification and acquisition at 94°C for 15 s, 58°C for 30 s, and 72°C for 30 s. The CFX Manager software was used to determine the copy number of the target DNA in the qPCR samples by plotting the data against the standard curve generated in parallel using known copy numbers of pNL43.

Isolation of HIV-1 cytoplasmic PICs and measurement of integration activity *in vitro.* The target cells (10×10^6) were spinoculated with virus stocks at $480 \times g$ for 2 h at 25°C. The spinoculated cells were collected and pelleted by centrifugation, and after removing the supernatants, were resuspended in new RPMI complete medium and cultured for 5 h at 37°C. The HIV-1 PICs were then isolated from the cell samples using a published protocol[58] with modifications that included two washes with 1 mL of buffer $K^{-/-}$ followed by cell resuspension in 0.5 mL of ice-cold buffer $K^{+/+}$. *In vitro* integration assays of cytoplasmic PICs were performed as described previously[58]. The first-round of the nested PCR, which selectively amplifies the integrated viral DNA-target DNA junctions, included one-tenth volume of the purified DNA from the in vitro integration reaction and the primers 5’-GTGCGCGCTTCAGCAAG-3’ and 5’-CACTGACCCTCAGCAATCTTA-3’, and was carried out at: 95°C for 5 min; followed by 23 cycles at 94°C for 30 s, 55°C for 30 s, and 72 °C for 4 min, and a final extension at 72°C for 10 min. The second-round qPCR included one-tenth volume of the first-round PCR products, 1X iTaq Universal Probe Supermix, primers 5′-TCTGGCTAACTAGGGAACCCA-3′ and 5′-CTGACTAAAAGGGTCTGAGG-3′, and the TaqMan probe 5′-[6-FAM]-

TTAAGCCTCAATAAAGCTTGCCTTGAGTGC-[TAMRA]-3′, and was carried out at: 95°C for 3 min, 39 cycles of amplification and acquisition at 94°C for 15 s, 58°C for 30 s, and 72°C for 30 s. To determine the PIC-associated viral DNA copy numbers, PICs was deproteinized, extracted with phenol-chloroform, subjected to ethanol precipitation, and one-tenth volume of the resulting purified DNA was directly used in qPCR designed to quantify the reverse transcription products. The CFX Manager software was used to determine the copy number of the target DNA in the qPCR samples by plotting the data against the standard curve that was generated in parallel using known copy numbers of pNL43 plasmid.

Sequence analysis of junctions of 2-LTR circles from HIV-1-infected target cells. A nested PCR strategy was used to amplify the junction of the 2-LTR circles in HIV-1-infected cells. The first-round PCR included 500 ng of total DNA from HIV-1-infected cells and the primers 2-LTR Forward-M (5′-AGCCTGGGAGCTCTCTGGCTAAC-3′) and 2-LTR Reverse-M (5′-AGCCTTGTGTGTGGTAGATCCAC-3′) and was carried out at: 98°C for 2 min, 30 cycles of amplification at 98°C for 10 s, 71°C for 1 min, and 72°C for 15 s, and a final extension at 72°C for 10 min. The second-round PCR included 1/10th volume of the first-round PCR products and the primers MH535 (5′-AACTAGGGAACCCACTGCTTAAG-3′) and MH536 (5′-TCCACAGATCAAGGATATCTTGTC-3′), and was carried out under the following thermocycling conditions: 95°C for 5 min, 25 cycles of amplification at 95°C for 15 s, 58°C for 1 min, and 72°C for 30 s, and a final extension at 72°C for 10 min. The resulting PCR amplicon was gel-purified and ligated to pGEMT-Easy vector (Promega), as per the manufacturer-recommended protocol. The sequence of the 2-LTR junction DNA inserts in the recombinant plasmids was determined by Sanger DNA sequencing using primers flanking the cloning site, and multiple alignment was used to analyze the sequence data.

SUPPLEMENTARY FIGURE LEGENDS

Figure S1. Electrostatic potential surfaces of the wildtype and three mutant hexamers. The CA hexamers are shown in molecular surface representations. The magnitude of the electrostatic potential is represented by the colors on the molecular surface. The regions of mutated residues are highlighted, indicating the electrostatic potential changes after residue substitutions.

Figure S2. Ion occupancies of CA hexamers. **(A)** Ion occupancies of four CA hexamer systems from CIONIZE. Occupancy of Na and Cl ion is in yellow and cyan respectively and the isovalue of the density map shown is 0.01 for both ions. **(B)** Relative chloride ion occupancy differences of three mutant hexamers, with respect to a selected reference system. The Cl^- ion occupancies of the RK hexamer with reference to WT hexamer, RKLM hexamer to RK hexamer, and RKLMSA hexamer to RKLM hexamer, are shown from left to right. The occupancies in blue represent chloride ion occupancy present in the test system but not in the reference system. Occupancies in lime representing ions present in the reference system but not in the test system.

Figure S3. Effect of KK10-linked CTL escape mutation R264K on the integration activity in vitro of cytoplasmic PICs**.** Integration activity in vitro and the viral DNA content of the WT and RK cytoplasmic PICs isolated from SupT1 cells spinoculated with the respective viruses for 2 hours at 25°C and cultured for 5 hours at 37°C were determined as described in Materials and Methods. **(A)** Integration activity of the cytoplasmic PICs, i.e., the copy number of viral DNAs that were integrated into the target DNA during the in vitro integration assay, as measured by qPCR and calculated using a standard graph of known copy numbers of HIV-1 molecular clone. **(B)** Percentage integration activity plotted relative to WT. **(C)** Copy number of viral DNA content in the cytoplasmic PICs as measured by qPCR and calculated using a standard graph of known copy numbers of HIV-1 molecular clone. **(D)** Percentage viral DNA content plotted relative to WT cytoplasmic PICs. **(E)** Ratio of the copy number of integrated viral DNAs to the corresponding PIC-associated viral DNAs plotted as percentage relative to WT. Data shown are representative of three independent experiments, with error bars representing the SEMs.

Figure S4. Assessment of the effect of KK10-linked CTL escape mutations on the integrity of the termini of the reverse transcription products (viral DNAs). Junctions of the 2-LTR circles present in the total DNA isolated from Jurkat cells spinoculated with the WT or mutant viruses for 2 hours at 25°C and cultured for 24 hours at 37°C were amplified by a nested PCR strategy, cloned into a plasmid vector, and the DNA sequences determined by Sanger sequencing were aligned and analyzed as described in Materials and Methods. Shown are the 2-LTR circle junction sequences from cells inoculated with **(A)** WT virus, **(B)** R264K virus, and **(C)** R264K/L268M/S173A virus. The 3'U5 sequence is in blue, the 5'U3 sequence is in red, the unprocessed terminal dinucleotides (GT and AC) are in black, the deletions within the 3'U5 or 5'U3 region are indicated by dashes, and the DNA insertions are in magenta. The insertion sequences highlighted in yellow or blue correspond to the PPT or PBS regions, respectively.

SUPPLEMENTARY FIGURES

Figure S1

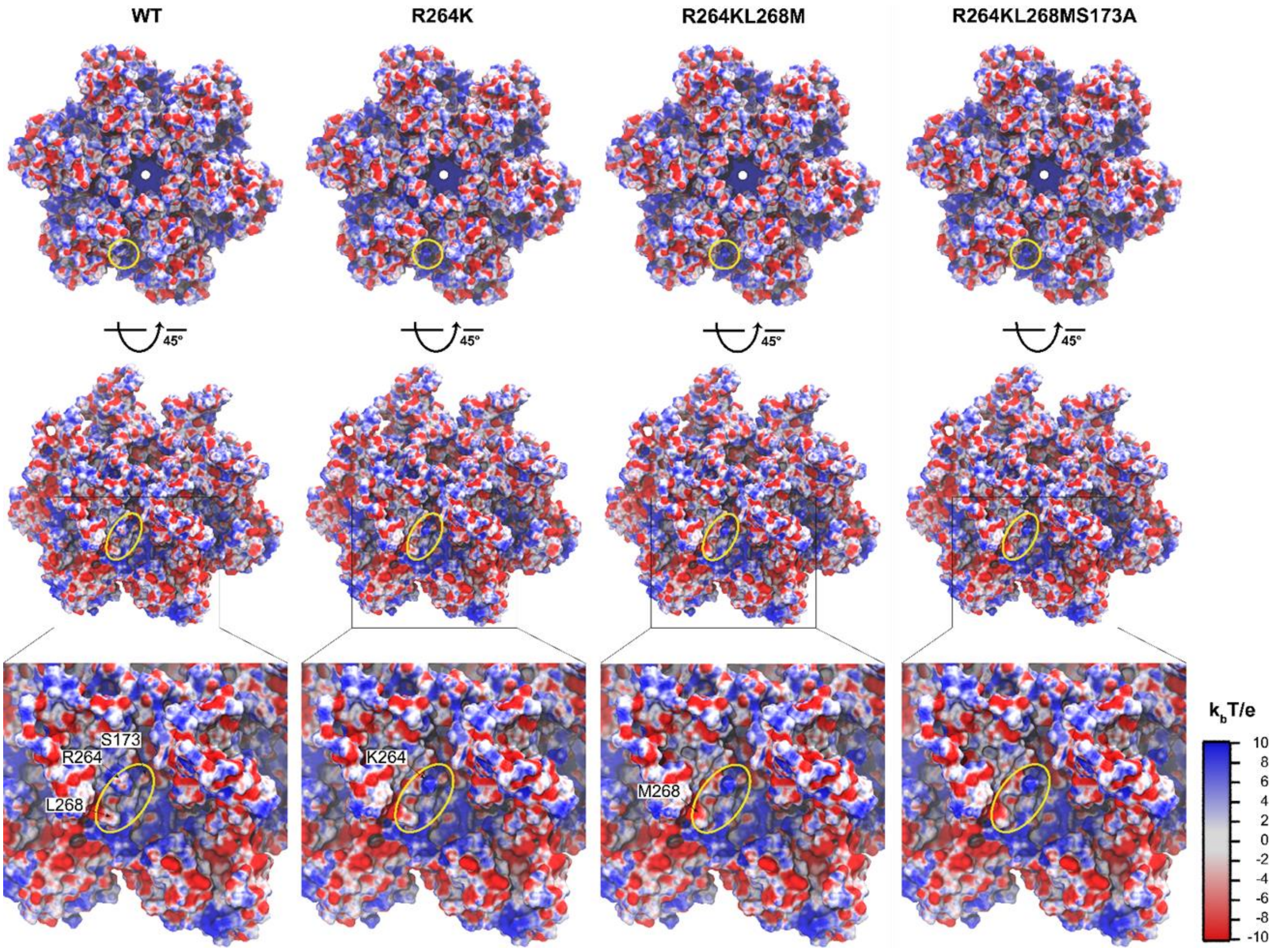

Figure S2

(A)

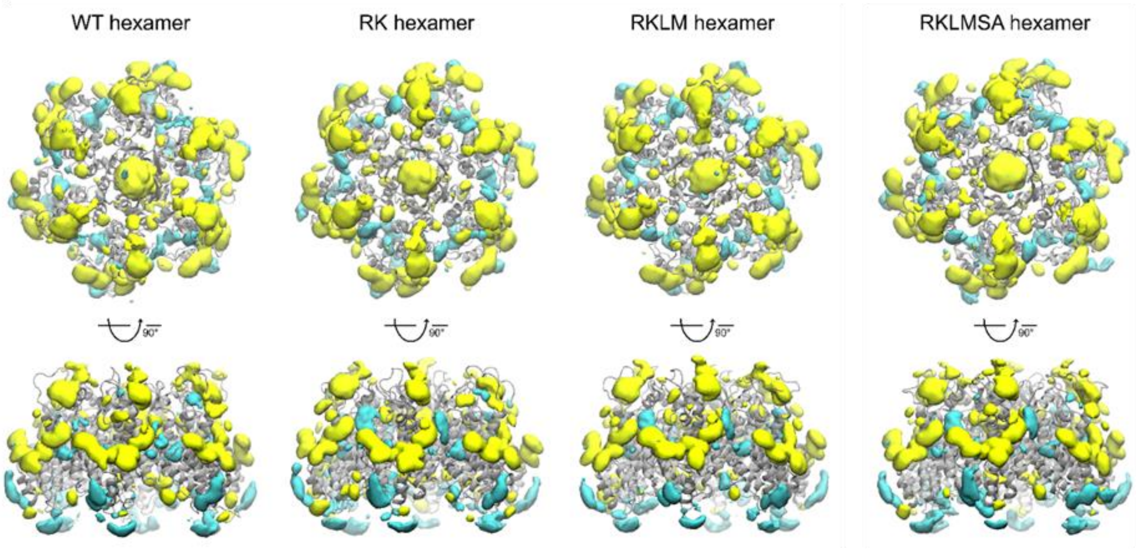
WT hexamer
RK hexamer
RKLM hexamer
RKLMSA hexamer
90°
90°
90°
90°

(B)

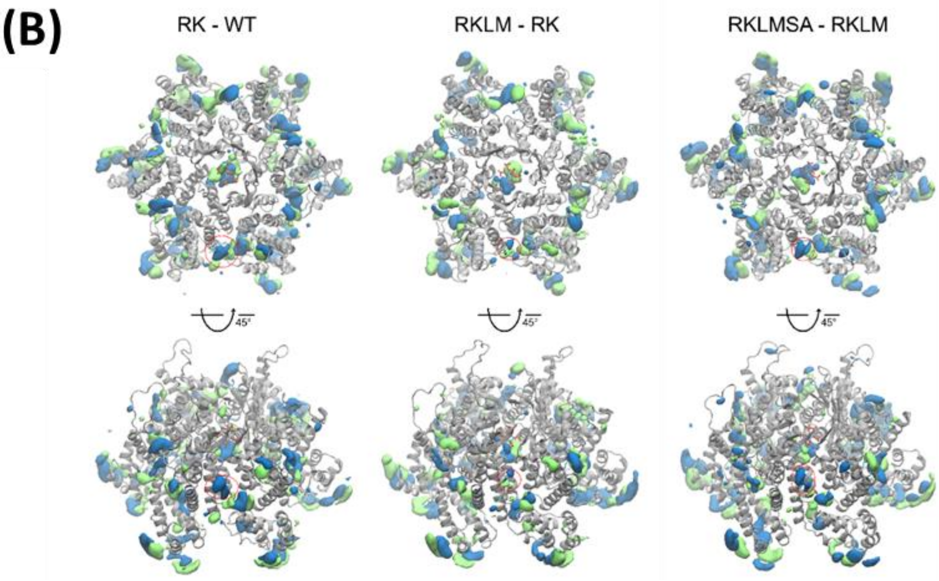
RK - WT
RKLM - RK
RKLMSA - RKLM
45°
45°
45°

Figure S3

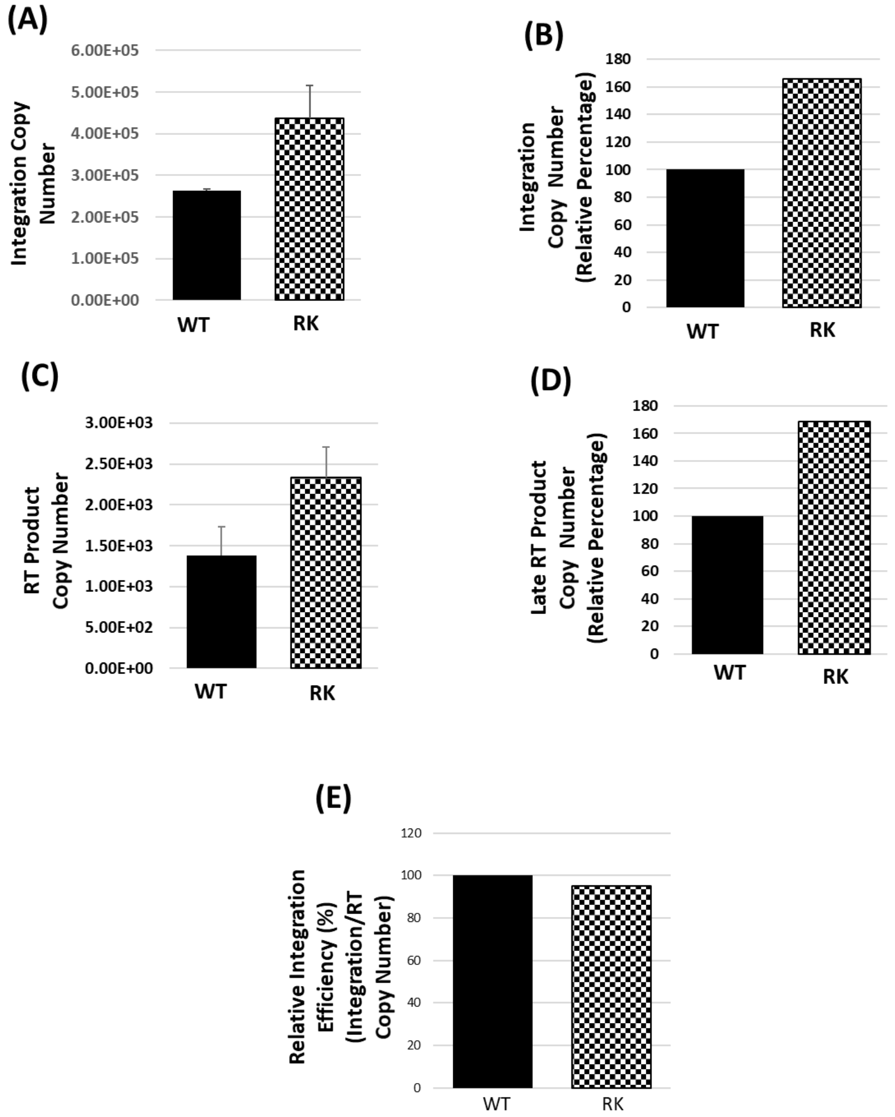
(A)
Integration Copy Number
6.00E+05
5.00E+05
4.00E+05
3.00E+05
2.00E+05
1.00E+05
0.00E+00
WT
RK
(B)
Integration Copy Number (Relative Percentage)
180
160
140
120
100
80
60
40
20
0
WT
RK
(C)
RT Product Copy Number
3.00E+03
2.50E+03
2.00E+03
1.50E+03
1.00E+03
5.00E+02
0.00E+00
WT
RK
(D)
Late RT Product Copy Number (Relative Percentage)
180
160
140
120
100
80
60
40
20
0
WT
RK
(E)
Relative Integration Efficiency (%) (Integration/RT Copy Number)
120
100
80
60
40
20
0
WT
RK

Figure S4

(A)

#	Right LTR Sequence	JUNCTION SEQUENCE<br>Terminal nucleotides GT/U5 and AC/U3<br>Insertion Sequence	Left LTR Sequence
1	TAGTCAGTGTGGAAAATCTCTAGCA	GTAC	TGGAAGGGCTAATTCACTCCCAAA
2	TAGTCAGTGTGGAAAATCTCTAGCA	GTAC	TGGAAGGGCTAATTCACTCCCAAA
3	TAGTCAGTGTGGAAAATCTCTAGCA	GTAC	TGGAAGGGCTAATTCACTCCCAAA
4	TAGTCAGTGTGGAAAATCTCTAGCA	GTAC	TGGAAGGGCTAATTCACTCCCAAA
5	TAGTCAGTGTGGAAAATCTCTAGCA	GTAC	TGGAAGGGCTAATTCACTCCCAAA
6	TAGTCAGTGTGGAAAATCTCTAGCA	GTAC	TGGAAGGGCTAATTCACTCCCAAA
7	TAGTCAGTGTGGAAAATCTCTAGCA	GTAC	TGGAAGGGCTAATTCACTCCCAAA
8	TAGTCAGTGTGGAAAATCTCTAGCA	GTAC	TGGAAGGGCTAATTCACTCCCAAA
9	TAGTCAGTGTGGAAAATCTCTAGCA	GTAC	TGGAAGGGCTAATTCACTCCCAAA
10	TAGTCAGTGTGGAAAATCTCTAGCA	GTAC	TGGAAGGGCTAATTCACTCCCAAA
11	TAGTCAGTGTGGAAAATCTCTAGCA	GTAC	TGGAAGGGCTAATTCACTCCCAAA
12	TAGTCAGTGTGGAAAATCTCTAGCA	GTAC	TGGAAGGGCTAATTCACTCCCAAA
13	TAGTCAGTGTGGAAAATCTCTAGCA	GTAC	TGGAAGGGCTAATTCACTCCCAAA
14	TAGTCAGTGTGGAAAATCTCTAGCA	GTAC	TGGAAGGGCTAATTCACTCCCAAA
15	TAGTCAGTGTGGAAAATCTCTAGCA	GTAC	TGGAAGGGCTAATTCACTCCCAAA
16	TAGTCAGTGTGGAAAATCTCTAGCA	G-AC	TGGAAGGGCTAATTCACTCCCAAA
17	TAGTCAGTGTGGAAAATCTCTAGCA	G-AC	TGGAAGGGCTAATTCACTCCCAAA
18	TAGTCAGTGTGGAAAATCTCTAGCA	G-AC	TGGAAGGGCTAATTCACTCCCAAA
19	TAGTCAGTGTGGAAAATCTCTAGCA	G-AC	TGGAAGGGCTAATTCACTCCCAAA
20	TAGTCAGTGTGGAAAATCTCTAGCA	G-AC	TGGAAGGGCTAATTCACTCCCAAA
21	TAGTCAGTGTGGAAAATCTCTAGCA	G-AC	TGGAAGGGCTAATTCACTCCCAAA
22	TAGTCAGTGTGGAAAATCTCTAGCA	G-AC	TGGAAGGGCTAATTCACTCCCAAA
23	TAGTCAGTGTGGAAAATCTCTAGCA	G-AC	TGGAAGGGCTAATTCACTCCCAAA
24	TAGTCAGTGTGGAAAATCTCTAGCA	G-AC	TGGAAGGGCTAATTCACTCCCAAA
25	TAGTCAGTGTGGAAAATCTCTAGCA	G-AC	TGGAAGGGCTAATTCACTCCCAAA
26	TAGTCAGTGTGGAAAATCTCTAGCA	G-AC	TGGAAGGGCTAATTCACTCCCAAA
27	TAGTCAGTGTGGAAAATCTCTAGCA	G-AC	TGGAAGGGCTAATTCACTCCCAAA
28	TAGTCAGTGTGGAAAATCTCTAGCA	G-AC	TGGAAGGGCTAATTCACTCCCAAA
29	TAGTCAGTGNGGAAAATCTNTAGCA	G-AC	TGGAAGGGCTAATTCACTCCCAAA
30	TAGTCAGTGTGGAAAATCTCTAGCA	G-AC	TGGAAGGGCTAATTCACTCCCAAA
31	TAGTCAGTGTGGAAAATCTCTAGCA	G-AC	TGGAAGGGCTAATTCACTCCCAAA
32	TAGTCAGTGTGGAAAATCTCTAGCA	G-AC	TGGAAGGGCTAATTCACTCCCAAA
33	TAGTCAGTGTGGAAAATCTCTAGCA	G-AC	TGGAAGGGCTAATTCACTCCCAAA
34	TAGTCAGTGTGGAAAATCTCTAGC-	G--C	TGGAAGGGCTAATTCACTCCCAAA
35	TAGTCAGTGTGGAAAATC-------	---C	TGGAAGGGCTAATTCACTCCCAAA
36	TAGTCAGTGTGGAAAATCTCTAGCA	----	-GGAAGGGCTAATTCACTCCCAAA
37	TAGTCAG------------------	----	TGGAAGGGCTAATTCACTCCCAAA
38	TAGTCAGTGTGGAAAATCTCTAGCA	----	-----GGGCTAATTCACTCCCAAA
39	TAGTCAGTGTGGAAAATCTCTAGCA	----	------GGCTAATTCACTCCCAAA
40	TAGTCAGTGTGGAAAATCTCTAGCA	----	--------CTAATTCACTCCCAAA
41	TAGTCAGTGTGGAANAT--------	----	----AGGGCTAANTCACTCCCAAA
42	TAGTCAGTGTGGAAAATCTCTAGCA	GTT--	---AAGGGCTAATTCACTCCCAAA
43	TAGTCAGTGTGGAAAATCTCTAGCA	GTCAC	TGGAAGGGCTAATTCACTCCCAAA
44	TAGTCAGTGTGGAAAATCTCTAGCA	G-CAC	TGGAAGGGCTAATTCACTCCCAAA
45	TAGTCAGTGTGGAAAATCTCTAGCA	GTGAC	TGGAAGGGCTAATTCACTCCCAAA
46	TAGTCAGTGTGGAAAATCTCTAGCA	G-AAC	TGGAAGGGCTAATTCACTCCCAAA
47	TAGTCAGTGTGGAAAATCTCTAGCA	GTAAC	TGGAAGGGCTAATTCACTCCCAAA
48	TAGTCAGTGTGGAAAATCTCTAGCA	GTC-C	TGGAAGGGCTAATTCACTCCCAAA
49	TAGTCAGTGTGGAAAATCTCTAGC-	--TAC	TGGAAGGGCTAATTCACTCCCAAA
50	TAGTCAGTGTGGAAAATCTCTAGCA	GTA--	---AAGGGCTAATTCACTCCCAAA
51	TAGTCAGTGTGGAAAATCTCTAGCA	GTA--	TGGAAGGGCTAATTCACTCCCAAA
52	TAGTCAGTGTGGAAAATCTCTAGCA	GTGGAC	TGGAAGGGCTAATTCACTCCCAAA
53	TAGTCAGTGTGGAAAATCTCTAGCA	GTGTAC	TGGAAGGGCTAATTCACTCCCAAA
54	TAGTCAGTGTGGAAAATCTCTAGCA	GTCGAC	TGGAAGGGCTAATTCACTCCCAAA
55	TAGTCAGTGTGGAAAATCTCTAGCA	GTTA--	---AAGGGCTAATTCACTCCCAAA
56	TAGTCAGTGTGGAAAATCTCTAGCA	GTCAAC	TGGAAGGGCTAATTCACTCCCAAA
57	TAGTCAGTGTGGAAAATCTCTAGCA	GTGTAC	TGGAAGGGCTAATTCACTCCCAAA
58	TAGTCAGTGTGGAAAATCTNTAGCA	GTAAAC	TGGAAGGGCTAATTCACTCCCAAA
59	TAGTCAGTGTGGAAAATCNCTAGCA	GTCAAC	TGGAAGGGCTAATTCACTCCCAAA
60	TAGTCAGTGTGGAAAATCTCTAGCA	GTGTAC	TGGAAGGGCTAATTCACTCCCAAA
61	TAGTCAGTGTGGAAAATCTCTAGCA	GTCAAC	TGGAAGGGCTAATTCACTCCCAAA
62	TAGTCAGTGTGGAAAATCTCTAGCA	GTTT--	---AAGGGCTAATTCACTCCCAAA
63	TAGTCAGTGTGGAAAATC-------	--AG--	-GGAAGGGCTAATTCACTCCCAAA
64	TAGTCAGTGTGGAAAATCTCTAG--	--TTG--	TGGAAGGGCTAATTCACTCCCAAA
65	TAGTCAGTGTGGAAAATCTCTAGCA	GTCCCAC	TGGAAGGGCTAATTCACTCCCAAA
66	TAGTCAGTGTGGAAAATCTCTAGCA	GTACAAC	TGGAAGGGCTAATTCACTCCCAAA
67	TAGTCAGTGTGGAAAATCTCTAGCA	GTTTAAC	TGGAAGGGCTAATTCACTCCCAAA
68	TAGTCAGTGTGGAAAATCTCTAGCA	GTCCCAC	TGGAAGGGCTAATTCACTCCCAAA
69	TAGTCAGTGTGGAAAATCTCTAGCA	GTTGGCAC	TGGAAGGGCTAATTCACTCCCAAA
70	TAGTCAGTGTGGAAAATCTCTAGCA	GTGGGGAC	TGGAAGGGCTAATTCACTCCCAAA
71	TAGTCAGTGTGGAAAATCTCTAGCA	--AGGGAC	TGGAAGGGCTAATTCACTCCCAAA
72	TAGTCAGTGTGGAAAATCTCTAGCA	GTCCAT--	TGGAAGGGCTAATTCACTCCCAAA
73	TAGTCAGTGTGGAAAATCTCTAGCA	G-GAACAAC	TGGAAGGGCTAATTCACTCCCAAA
74	TAGTCAGTGTGGAAAATCTCTAGCA	--GGGAGAC	TGGAAGGGCTAATTCACTCCCAAA
75	TAGTCAGTGTGGAAAATCTCTAGCA	GTTCTCT--	TGGAAGGGCTAATTCACTCCCAAA
76	TAGTCAGTGTGGAAAATCTCTAGCA	GTAGGACAC	TGGAAGGGCTAATTCACTCCCAAA
77	TAGTCAGTGTGGAAAATCTCTAGCA	GTGAGGAAC	TGGAAGGGCTAATTCACTCCCAAA
78	TAGTCAGTGTGG-------------	--GGGGGGAC	TGGAAGGGCTAATTCACTCCCAAA
79	TAGTCAGTGTGGAAAATCTCTAGCA	GTATCCAA--	---AATGGCTAATTCACTCCCAAA
80	TAGTCAGTGTGGAAAAT--------	--GACTGTCAC	TGGAAGGGCTAATTCACTCCCAAA
81	TAGTCAGTGTGGAAAATCTNTAGCA	--ACTCTTCAC	TGGAAGGGCTAATTCACTCCCAAA
82	TAGTCAGTGTGGAAAAT--------	--GACTGTCAC	TGGAAGGGCTAATTCACTCCCAAA
83	TAGTCAGTGTGGAAAATCTCTAGCA	GTCTAACAAATAC	TGGAAGGGCTAATTCACT-CCAAA
84	TAGTCAGTGTGGAAAATCTCTAGCA	GTGGTTCCGTAT--	TGGAATGGCTAATTCACTCCCAAA
85	TAGTCAGTGTGGAAAATCTCTAGCA	GTGCCCATCCTT--	TGGAAGGGCTAATTCACTCCCAAA
86	TAGTCAGTGTGGAAAATCTCTAGCA	--AAAAGGGGGGAC	TGGAAGGGCTAATTCACTCCCAAA
87	TAGTCAGTGTGGAAAATCTCTAGCA	GTGGCCCAGGGGTAC	TGGAAGGGCTAATTCACTCCCAAA
88	TAGTCAGTGTGGAAAATCTCTAGCA	G-CTCACGGCCGCGAC	TGGAAGGGCTAATTCACTCCCAAA
89	TAGTCAGTGTGGAAAATCTCTAGCA	GTGGCGCCTGAACAGGGACAC	TGGAAGGGCTAATTCACTCCCAAA
90	TAGTCAGTGTGGAAAATCTCTAGCA	GTGGCGCCCGAACAGGGACAC	TGGAAGGGCTAATTCACTCCCAAA
91	TAGTCAGTGTGGAAAATCTCTAGCA	GTGGCGCCCGAACAGGGACAC	TGGAAGGGCTAATTCACTCCCAAA
92	TAGTCAGTGTGGAAAATCTCTAGCA	GTGGCGCCCGAACAGGGACAC	TGGAAGGGCTAATTCACTCCCAAA
93	TAGTCAGTGTGGAAAATCTCTAGCA	GTGGCGCCCGAACAGGGACAC	TGGAAGGGCTAATTCACTCCCAAA
94	TAGTCAGTGTGGAAAATCTCTAGCA	GTGCTGCCCGGNCTAGGTCAC	TGNNAGGGCTAATTCACTCCCNNN
95	TAGTCAGTGTGGAAAATCTCTAGCA	GTGGCGCCCGAACAGGGACTTGA--	TGGAAGGGCTAATTCACTCCCAAA
96	TAGTCAGTGTGGAAAATCTCTAGCA	GTTAATTTTAAAAGAAAAGGGGGGAC	TGGAAGGGCTAATTCACTCCCAAA
97	TAGTCAGTGTGGAAAATCTCTAGCA	GTTGGTGGCCCACTTCTGGTACCATAC	TGGAAGGGCTAATTCACTCCCAAA
98	TAGTCAGTGTGGAAAATCTCTAGCA	GTAAATTTTTAAAAGAAAAGGGGGGAC	TGGAAGGGCTAATTCACTCCCAAA
99	TAGTCAGTGTGGAAAATCTCTAGCA	GTTGGTGACCCCACGTTGGGCGCCAAC	TGGAAGGGCTAATTCACTCCCAAA
100	TAGTCAGTGTGGAAAATCTCTAGCA	GTGGCGCCCGAACAGGGACTTGAAAGCGAAAGT--	TGGAAGGGCTAATTCACTCCCAAA
101	TAGTCAGTGTGGAAAATCTCTAGCA	GTTTGGTGGACCGCACGGGGTCTCCCCGCGCCAGGTAC	TGGAAGGGCTAATTCACTCCCAAA
102	TAGTCAGTGTGGAAAATCTCTAGCA	--TAGATCTTAGCCACTTTTTAAAAGAAAAGGGGGGAC	TGGAAGGGCTAATTCACTCCCAAA
103	TAGTCAGTGTGGAAAATCTCTAGCA	GTATTGTGTGCATCAAAGGATAGATGTAAAAGACACCAC	TGGAAGGGCTAATTCACTCCCAAA
104	TAGTCAGTGTGGAAAATCTCTAGCA	GTGGCGCCCGAACAGGGACGTTTTCCAGTCACACCTCAGGTACCTTTAAGACCAATGACT<br>TACAAGGCAGCTGTAGATCTTAGCCACTTTTTAAAAGAAAAGGGGGGAC	TGGAAGGGCTAATTCACTCCCAAA

Figure S4

(B)

#	Right LTR Sequence	JUNCTION SEQUENCE<br>Terminal nucleotides GT/U5 and AC/U3<br>Insertion Sequence	Left LTR Sequence
1	TAGTCAGTGTGGAAAATCTCTAGCA	GTAC	TGGAAGGGCTAATTCACTCCCAAA
2	TAGTCAGTGTGGAAAATCTCTAGCA	GTAC	TGGAAGGGCTAATTCACTCCCAAA
3	TAGTCAGTGTGGAAAATCTCTAGCA	GTAC	TGGAAGGNCTAATTCACTCCCAAA
4	TAGTCAGTGTGGAAAATCTCTAGCA	GTAC	TGGAAGGGCTAATTCACTCCCAAA
5	TAGTCAGTGTGGAAAATCTCTAGCA	GTAC	TGGAAGGGCTAATTCACTCCCAAA
6	TAGTCAGTGTGGAAAATCTCTAGCA	GTAC	TGGAAGGGCTAATTCACTCCCAAA
7	TAGTCAGTGTGGAAAATCTCTAGCA	GTAC	TGGAAGGGCTAATTCACTCCCAAA
8	TAGTCAGTGTGGAAAATCTCTAGCA	G-AC	TGGAAGGGCTAATTCACTCCCAAA
9	TAGTCAGTGTGGAAAATCTCTAGCA	G-AC	TGGAAGGGCTAATTCACTCCCAAA
10	TAGTCAGTGTGGAAAATCTCTAGCA	G-AC	TGGAAGGGCTAATTCACTCCCAAA
11	TAGTCAGTGTGGAAAATCTCTAGCA	G-AC	TGGAAGGGCTAATTCACTCCCAAA
12	TAGTCAGTGTGGAAAATCTCTAGCA	G-AC	TGGAAGGGCTAATTCACTCCCAAA
13	TAGTCAGTGTGGAAAATCTCTAGCA	G-AC	TGGAAGGGCTAATTCACTCCCAAA
14	TAGTCAGTGTGGAAAATCTCTAGCA	G-AC	TGGAAGGGCTAATTCACTCCCAAA
15	TAGTCAGTGTGGAAAATCTCTAGCA	G-AC	TGGAAGGGCTAATTCACTCCCAAA
16	TAGTCAGTGTGGAAAATCTCTAGCA	G-AC	TGGAAGGGCTAATTCACTCCCAAA
17	TAGTCAGTGTGGAAAATCTCTAGCA	G-AC	TGGAAGGGCTAATTCACTCCCAAA
18	TAGTCAGTGTGGAAAATCTCTAGCA	GT-C	TGGAAGGGCTAATTCACTCCCAAA
19	TAGTCAGTGTGGAAAATCTCTAGCA	G-AC	TGGAAGGGCTAATTCACTCCCAAA
20	-------------------------	--AC	TGGAAGGGCTAATTCACTCCCAAA
21	TAGTCAGTGTGGAAAATCTCTAGCA	--AC	TGGAAGGGCTAATTCACTCCCAAA
22	TAGTCAGTGTGGAAAATCTCTAGCA	GT--	----------AATTCACTCCCAAA
23	TAGTCAGTGTGGAAAATCTCTAG--	--AC	TGGAAGGGCTAATTCACTCCCAAA
24	TAGTCAGTGTGGAAAA---------	--AC	TGGAAGGGCTAATTCACTCCCAAA
25	TAGTCAGTGTGGAAAATCTCTAGCA	GT--	TGGAAGGGCTAATTCACTCCCAAA
26	TAGTCAGTGTGGAAAATCTCTAGCA	GT--	------------------------
27	TAGTCAGTGTGGAAAATCTCTAGCA	----	--------------CACTCCCAAA
28	TAGTCAGTGTGGAAAATCTCTAGCA	----	------------TTCACTCCCAAA
29	TAGTCAGTGTGGAAAATCTCTAGCA	----	---AAGGGCTAATTCACTCCCAAA
30	TAGTCA-------------------	----	-----GGGCTAATTCACTCCCAAA
31	TAGTCAGTGTGGAAAATCTCTAGCA	----	--------CTAATTCACTCCCAAA
32	-------------------------	----	TGGAAGGGCTAATTCACTCCCAAA
33	TAGTCAGTGTGGAAAATCTCTAGCA	----	----------------CTCCCAAA
34	TAGTCAGTGTGGAAAATCTCTAGCA	----	--------CTAATTCACTCCCAAA
35	TAGTCAGTGTGGAAAATCTCTAGCA	----	----AGGGCTAATTCACTCCCAAA
36	TAGTCAGTGTG--------------	----	TGGAAGGGCTAATTCACTCCCAAA
37	TAGTCAGTGTGGAAAATCTCTAGCA	----	------------------------
38	-------------------------	----	------------------------
39	TAGTCAGTGTGGAAAATCTCTAGCA	----	---AAGGGCTAATTCACTCCCAAA
40	TAGTCAGTGTGGAAAATCTCTAGCA	----	--------CTAATTCACTCCCAAA
41	TAGTCAGTGTGGAAAATCTCTAGCA	GTAAC	TGGAAGGGCTAATTCACTCCCAAA
42	TAGTCAGTGTGGAAAATCTCTAGCA	G-GAC	TGGAAGGGCTAATTCACTCCCAAA
43	TAGTCAGTGTGGAAAATCTCTAGCA	GTGAC	TGGAAGGGCTAATTCNCTCCCAAA
44	TAGTCAGTGTGGAAAATCTCTAGCA	GTG--	TGGAAGGGCTAATTCACTCCCAAA
45	TAGTCAGTGTGGAAAATCTCTAGCA	GAGGAC	TGGAAGGGCTAATTCACTCCCAAA
46	TAGTCAGTGTGGAAAATCTCTAGCA	GTGG-C	TGGAAGGGCTAATTCACTCCCAAA
47	TAGTCAGTGTGGAAAAT--------	--AGAC	TGGAAGGGCTAATTCACTCCCAAA
48	TAGTCAGTGTGGAAAATCTCTAGCA	GTACTAC	TGGAAGGGCTAATTCACTCCCAAA
49	TAGTCAGTGTGGAAAATCTCTAGCA	GTCCAC	TGGAAGGGCTAATTCACTCCCAAA
50	TAGTCAGTGTGG-------------	--GGAC	TGGAAGGGCTAATTCACTCCCAAA
51	TAGTCAGTGTGGAAAATCTCTAGCA	GGGGGAC	TGGAAGGGCTAATTCACTCCCAAA
52	TAGTCAGTGTGGAAAATCTCTAGCA	GTGTC-C	TGGAAGGGCTAATTCACTCCCAAA
53	TAGTCAGTGTGGAAAATCTCTAGCA	--GAG--	TGGAAGGGCTAATTCACTCCCAAA
54	TAGTCAGTGTGGAAAATCTCTAGCA	G-GCGGAC	TGGAAGGGCTAATTCACTCCCAAA
55	TAGTCAGTGTGGAAAATCTCTAGCA	--GGGGAC	TGGAAGGGCTAATTCACTCCCAAA
56	TAGTCAGTGTGGAAAATCTCTAGCA	--GCCAAC	TGGAAGGGCTAATTCACTCCCAAA
57	TAGTCAGTGTGGAAAATCTCTAGCA	--GGGGG--	-----------ATTCACTCCCAAA
58	TAGTCAGTGTGGAAAATCTCTAGCA	GTAGCAG--	TGGAAGGGCTAATTCACTCCCAAA
59	TAGTCAGTGTGGAAAATCTCTAGCA	--AGGAGAC	TGGAAGGGCTAATTCACTCCCAAA
60	TAGTCAGTGTGGAAAATCTCTAGCA	GTTCCCCAC	TGGAAGGGCTAATTCACTCCCAAA
61	TAGTCAGTGTGGAAAATCTCTAG--	--AGGGAGAC	TGGAAGGGCTAATTCACTCCCAAA
62	TAGTCAGTGTGGAAAATCTCTAGCA	--GGGGGGAC	TGGAAGGGCTAATTCACTCCCAAA
63	TAGTCAGTGTGGAAAATCTCTAGCA	GTATTGGAGAC	TGGAAGGGCTAATTCACTCCCAAA
64	TAGTCAGTGTGGAAAATCTCTAGCA	GTGGCGCCC--	TGGAAGGGCTAATTCACTCCCAAA
65	TAGTCAGTGTGGAAAATCTCTAGCA	GTGGCGCCC--	TGGAAGGGCTAATTCACTCCCAAA
66	TAGTCAGTGTGGAAAATCTCTAGCA	GTGGCGCCC--	TGGAAGGGCTAATTCACTCCCAAA
67	TAGTCAGTGTGGAAAATCTCTAGCA	GTGGCGCCC--	TGGAAGGGCTAATTCACTCCCAAA
68	TAGTCAGTGTGGAAAATCTCTAGCA	GTGGCGCCC--	TGGAAGGGCTAATTCACTCCCAAA
69	TAGTCAGTGTGGAAAATCTCTAGCA	GTGGCGCCC--	TGGAAGGGCTAATTCACTCCCAAA
70	TAGTCAGTGTGGAAAATCTCTAGCA	GTGGCGCCC--	TGGAAGGGCTAATTCACTCCCAAA
71	TAGTCAGTGTGGAAAATCTCTAGCA	GTGGCGCCCGA--	TGGAAGGGCTAATTCACTCCCAAA
72	TAGTCAGTGTGGAAAATCTCTAGCA	GTGGCGCCCGAACAGGGACAC	TGGAAGGGCTAATTCACTCCCAAA
73	TAGTCAGTGTGGAAAATCTCTAGCA	GTGGCGCCCGAACAGGGACAC	TGGAAGGGCTAATTCACTCCCAAA
74	TAGTCAGTGTGGAAAATCTCTAGCA	GTGGCGCCCGAACAGGGACTAC	TGGAAGGGCTAATTCACTCCCAAA
75	TAGTCAGTGTGGAAAATCTCTAGCA	GTTGGTGCCGTGACTCGGATAC	TGGAAGGGCTAATTCACTCCCAAA
76	TAGTCAGTGTGGAAAATCTCTAGCA	GTGGCGCCCGAACAGGGACAAC	TGGAAGGGCTAATTCACTCCCAAA
77	TAGTCAGTGTGGAAAATCTCTAGCA	GTGGCGCCTGAACGGGGAGAC	TGGAAGGGCTAATTCACTCCCAAA
78	TAGTCAGTGTGGAAAATCTCTAGCA	GTGGCGCCCGAACAGGGACAC	TGGAAGGGCTAATTCACTCCCAAA
79	TAGTCAGTGTGGAAAATCTCTAGCA	GTTGGGGGCTCGTCCGGGATAC	TGGAAGGGCTAATTCACTCCCAAA
80	TAGTCAGTGTGGAAAATCTCTAGCA	GTATTTTAAAAGAAAAGGGGGGAC	TGGAAGGGCTAATTCACTCCCAAA
81	TAGTCAGTGTGGAAAATCTCTAGCA	GTGGCGCCCGAACAGGGACAGGAC	TGGAAGGGCTAATTCACTCCCAAA
82	TAGTCAGTGTGGAAAATCTCTAGCA	GTCATTTTAAAAGAAAAGGGGGGAC	TGGAAGGGCTAATTCACTCCCAAA
83	TAGTCAGTGTGGAAAATCTCTAGCA	G_AGGCGCCCGAACAGGGACATGAAGGGGGGAC	TGGAAGGGCTAATTCACTCCCAAA
84	TAGTCAGTGTGGAAAATCTCTAGCA	G-ATCTTAGCCACTTTTTAAAAGAAAAGGGGGGAC	TGGAAGGGCTAATTCACTCCCAAA
85	TAGTCAGTGTGGAAAATCTCTAGCA	GTATATAAAATTAGCAGGAAGATGGCCAGTAAAAAC	TGGAAGGGCTAATTCACTCCCAAA
86	TAGTCAGTGTGGAAAATCTCTAGCA	--AGATCTTAGCCACTTTTTAAAAGAAAAGGGGGGAC	TGGAAGGGCTAATTCACTCCCAAA
87	TAGTCAGTGTGGAAAATCTCTAGCA	--AGATCTTAGCCACTTTTTAAAAGAAAAGGGGGGAC	TGGAAGGGCTAATTCACTCCCAAA
88	TAGTCAGTGTGGAAAATCTCTAGCA	GTGGCGCCCGAACAGGGACTTGAAAGCGAAAGTAAA--	------GGCTAATTCACTCCCAAA
89	TAGTCAGTGTGGAAAATCTCTAGCA	--TAGATCTTAGCCACTTTTTAAAAGAAAAGGGGGGAC	TGGAAGGGCTAATTCACTCCCAAA
90	TAGTCAGTGTGGAAAATCTCTAGCA	GTGGCGCCCGAACAGGGACTTGAAAGCGAAAGTAAA--	---AAGGGCTAATTCACTCCCAAA
91	TAGTCAGTGTGGAAAATCTCTAGCA	GTGGCGCCCGAACAGGGACTTGAAAGCGAAAGTAAAGAC	TGGAAGGGCTAATTCACTCCCAAA
92	TAGTCAGTGTGGAAAATCTCTAGCA	GTGGCGCCCGAACAGGGACTTGAAAGCGAAAGTAAAGCAC	TGGAAGGGCTAATTCACTCCCAAA
93	TAGTCAGTGTGGAAAATCTCTAGCA	GTGGCGCCCGAACAGGGACTTGAAAGCGAAAGTAAAACCA--	---AAGGGCTAATTCACTCCCAAA
94	TAGTCAGTGTGGAAAATCTCTAGCA	GTCCCCCCTTTTCTTTTAAAAAGTGGCTAAGATCTACATTTTAC	TGGAAGGGCTAATTCACTCCCAAA
95	TAGTCAGTGTGGAAAATCTCTAGCA	GTCCCCCCTTTTCTTTTAAAAAGTGGCTAAGAAATAATTTTATAAC	TGGAAGGGCTAATTCACTCCCAAA
96	TAGTCAGTGTGGAAAATCTCTAGCA	--AAGGCAGCTGTGGATCTTAGCCACTTTTTAAAAGAAAAGGGGGGAC	TGGAAGGGCTAATTCACTCCCAAA
97	TAGTCAGTGTGGAAAATCTCTAGCA	--CAAGGCAGCTGTAGATCTTAGCCACTTTTTAAAAGAAAAGGGGGGAC	TGGAAGGGCTAATTCACTCCCAAA
98	TAGTCAGTGTGGAAAATCTCTAGCA	GTGGCGCCCGAACAGGGACTTGAAAGCGAAAGTAGAGCCAGAGGAGAT--	TGGAAGGGCTAATTCACTCCCAAA
99	TAGTCAGTGTGGAAAATCTCTAGCA	GTGGCGCCCGAACAGGGACTTGAAAGCGAAAGTAAAGCCGGAGGAGAT--	TGGAAGGGCTAATTCACTCCCAAA
100	TAGTCAGTGTGGAAAATCTCTAGCA	GTGGCGCCCGAACAGGGACTTGAAAGCGAAAGTAAAGCTTTTAAAAGAAAAGGGGGGAC	TGGAAGGGCTAATTCACTCCCAAA
101	TAGTCAGTGTGGAAAATCTCTAGCA	--CCAATGACTTACAAGGCAGCTGTAGATCTTAGCCACTTTTTAAAAGAAAAGGGGGGAC	TGGAAGGGCTAATTCACTCCCAAA
102	TAGTCAGTGTGGAAAATCTCTAGCA	--AGACCAATGACTTACAAGGCAGCTGTAGATCTTAGCCACTTTTTAAAAGAAAAGGGGGGAC	TGGAAGGGCTAATTCACTCCCAAA
103	TAGTCAGTGTGGAAAATCTCTAGCA	--AAGACCAATGACTTACAAGGCAGCTGTAGATCTTAGCCACTTTTTAAAAGAAAAGGGGGGAC	TGGAAGGGCTAATTCACTCCCAAA
104	TAGTCAGTGTGGAAAATCTCTAGCA	GTCTAAGACCAATGACTTACAAGGCAGCTGTAGATCTTAGCCACTTTTTAAAAGAAAAGGGGGGAC	TGGAAGGGCTAATTCACTCCCAAA
105	TAGTCAGTGTGGAAAATCTCTAGCA	--TTTAAGACCAATGACTTACAAGGCAGCTGTAGATCTTAACCACTTTTTAAAAGAAAAGGGGGGAC	TGGAAGGGCTAATTCACTCCCAAA
106	TAGTCAGTGTGGAAAATCTCTAGCA	GTGGCGCCCGAACAGGGACTTGAAAGCGAAAGTAAAGCCAGAGGAGATCTCTCGACGCAGGACTCGG--	TGGAAGGGCTAATTCACTCCCAAA

Figure S4

(C)

#	Right LTR Sequence	JUNCTION SEQUENCE<br>Terminal nucleotides GT/U5 and AC/U3<br>Insertion Sequence	Left LTR Sequence
1	TAGTCAGTGTGGAAAATCTCTAGCA	GTAC	TGGAAGGGCTAATTCACTCCCAAA
2	TAGTCAGTGTGGAAAATCTCTAGCA	GTAC	TGGAAGGGCTAATTCACTCCCAAA
3	TAGTCAGTGTGGAAAATCTCTAGCA	GTAC	TGGAAGGGCTAATTCACTCCCAAA
4	TAGTCAGCGTGGAAAATCTCTAGCA	GTAC	TGGAAGGGCTAATTCACTCCCAAA
5	TAGTCAGTGTGGAAAATCTCTAGCA	GTAC	TGGAAGGGCTAATTCACTCCCAAA
6	TAGTCAGTGTGGAAAATCTCTAGCA	GTAC	TGGAAGGGCTAATTCACTCCCAAA
7	TAGTCAGTGTGGAAAATCTCTAGCA	GTAC	TGGAAGGGCTAATTCACTCCCAAA
8	TAGTCAGTGTGGAAAATCTCTAGCA	GTAC	TGGAAGGGCTAATTCACTCCCAAA
9	TAGTCAGTGTGGAAAATCTCTAGCA	GTAC	TGGAAGGGCTAATTCACTCCCAAA
10	TAGTCAGTGTGGAAAATCTCTAGCA	GTAC	TGGAAGGGCTAATTCACTCCCAAA
11	TAGTCAGTGTGGAAAATCTCTAGCA	GTAC	TGGAAGGGCTAATTCACTCCCAAA
12	TAGTCAGTGTGGAAAATCTCTAGCA	GTAC	TGGAAGGGCTAATTCACTCCCAAA
13	TAGTCAGTGTGGAAAATCTCTAGCA	GTAC	TGGAAGGGCTAATTCACTCCCAAA
14	TAGTCAGTGTGGAAAATCTCTAGCA	G-AC	TGGAAGGGCTAATTCACTCCCAAA
15	TAGTCAGTGTGGAAAATCTCTAGCA	G-AC	TGGAAGGGCTAATTCACTCCCAAA
16	TAGTCAGTGTGGAAAATCTCTAGCA	GT-C	TGGAAGGGCTAATTCACTCCCAAA
17	TAGTCAGTGTGGAAAATCTCTAGCA	GT--	---AAGGGCTAATTCACTCCCAAA
18	TAGTCAGTGTGGAAAATCTCTAGCA	GT--	--GAAGGGCTAATTCACTCCCAAA
19	-------------------------	--AC	TGGAAGGGCTAATTCACTCCCAAA
20	-------------------------	--AC	TGGAAGGGCTAATTCACTCCCAAA
21	-------------------------	--AC	TGGAAGGGCTAATTCACTCCCAAA
22	TAGTCAGTGTGG-------------	--AC	TGGAAGGGCTAATTCACTCCCAAA
23	-------------------------	--AC	TGGAAGGGCTAATTCACTCCCAAA
24	TAGTCAGTGT---------------	--AC	TGGAAGGGCTAATTCACTCCCAAA
25	TAGTCAGTGT---------------	--AC	TGGAAGGGCTAATTCACTCCCAAA
26	TAGTCAGTGTGGAAAATCTCTAGCA	G--C	TGGAAGGGCTAATTCACTCCCAAA
27	TAGTCAGTGTGGAAAATCTCTAGCA	G--C	TGGAAGGGCTAATTCACTCCCAAA
28	TAGTCAGTGTGGAAAATCTCTAGCA	G---	TGGAAGGGCTAATTCACTCCCAAA
29	TAGTCAGTGTGGAAAATCTCTAGCA	G---	TGGAAGGGCTAATTCACTCCCAAA
30	TAGTCAGTGTGGAAAATCTCTAGCA	G---	TGGAAGGGCTAATTCACTCCCAAA
31	TAGTCAGTGTGGAAAATCTCTAGCA	G---	TGGAAGGGCTAATTCACTCCCAAA
32	-------------------------	---C	TGGAAGGGCTAATTCACTCCCAAA
33	TAGTCAGTGTGGAAAATCTCTAGCA	----	-----GGGCTAATTCACTCCCAAA
34	TAGTCAGTGTGGA------------	----	---AAGGGCTAATTCACTCCCAAA
35	TAGTCAGTGTGGA------------	----	---AAGGGCTAATTCACTCCCAAA
36	TAGTCAGTGTGGAAAATCTCT----	----	------------------------
37	TAGTCAGTGTGGAAAATCT------	----	-----------------TCCTAAA
38	-------------------------	----	-GGAAGGGCTAATTCACTCCCAAA
39	TAGTCAGTGTGGAAAATCTCTAGC-	----	-----GGGCTAATTCACTCCCAAA
40	TAGTCAGTGTGGAAAATCTCTAGCA	----	--------CTAATTCACTCCCAAA
41	TAGTCAGTGTGGAAAATCTCTAGC-	----	-----GGGCTAATTCACTCCCAAA
42	TAGTCAGTGTGGAAAATCTCTAGC-	----	-----GGGCTAATTCACTCCCAAA
43	TAGT---------------------	----	-----GGGCTAATTCACTCCCAAA
44	-------------------------	----	TGGAAGGGCTAATTCACTCCCAAA
45	-------------------------	----	-----GGGCTAATTCACTCCCAAA
46	TAGTCAGTGTGGAAAATCTCTAGCA	----	-GGAAGGGCTAATTCACTCCCAAA
47	-------------------------	----	TGGAAGGGCTAATTCACTCCCAAA
48	TAGTCAGTGTGGAAAATCTCTAGCA	GTCAC	TGGAAGGGCTAATTCACTCCCAAA
49	TAGTCAGTGTGGAAAATCTCTAGCA	GTCAC	TGGAAGGGCTAATTCACTCCCAAA
50	TAGTCAGTGTGGAAAATCTCTAGCA	GTGAC	TGGAAGGGCTAATTCACTCCCAAA
51	TAGTCAGTGTGGAAAATCTCTAGCA	GTGAC	TGGAAGGGCTAATTCACTCCCAAA
52	TAGTCAGTGTGGAAAATCTCTAGCA	--C-C	TGGAAGGGCTAATTCACTCCCAAA
53	TAGTCAGTGTGGAAAATCTCTAGCA	GTT--	TGGAAGGGCTAATTCACTCCCAAA
54	TAGTCAGTGTGGAAAATCTCTAGCA	GTTAC	TGGAAGGGCTAATTCACTCCCAAA
55	T------------------------	--CCAC	TGGAAGGGCTAATTCACTCCCAAA
56	TAGTCAGTGTGGAAAATCTCTAGCA	GTGGAC	TGGAAGGGCTAATTCACTCCCAAA
57	TAGTCAGTGTGGAAAATCTCTAGCA	GTATAC	TGGAAGGGCTAATTCACTCCCAAA
58	TAGTCAGTGTGGAAAATCTCTAGCA	GTGGAC	TGGAAGGGCTAATTCACTCCCAAA
59	TAGTCAGTGTGGAAAATCTCTAGCA	GTGGAC	TGGAAGGGCTAATTCACTCCCAAA
60	TAGTCAGTGTGGAAAATCTCTAGCA	G-GTAC	TGGAAGGGCTAATTCACTCCCAAA
61	TAGTCAGTGTGGAAAATCTCTAGCA	G-GTAC	TGGAAGGGCTAATTCACTCCCAAA
62	TAGTCAGTGTGGAAAATCTCTAGCA	GTAAAC	TGGAAGGGCTAATTCACTCCCAAA
63	TAGTCAGTGTGGAAAATCTCTAGCA	GTGAAC	TGGAAGGGCTAATTCACTCCCAAA
64	TAGTCAGTGTGGAAAATCTCTAGCA	GTAAAC	TGGAAGGGCTAATTCACTCCCAAA
65	TAGTCAGTGTGGAAAATCTCTAGCA	GTCTAC	TGGAAGGGCTAATTCACTCCCAAA
66	TAGTCAGTGTGGAAAATCTCTAGCA	GTGCAC	TGGAAGGGCTAATTCACTCCCAAA
67	TAGTCAGTGTGGAAAATCTCTAGCA	GTTCAC	TGGAAGGGCTAATTCACTCCCAAA
68	TAGTCAGTGTGGAAAATCTCTAGCA	--AGAC	TGGAAGGGCTAATTCACTCCCAAA
69	TAGTCAGTGTGGAAAATCTCTAGCA	GTAAAC	TGGAAGGGCTAATTCACTCCCAAA
70	TAGTCAGTGTGGAAAATCTCTAGCA	GTGCAC	TGGAAGGGCTAATTCACTCCCAAA
71	TAGT---------------------	--TAAC	TGGAAGGGCTAATTCACTCCCAAA
72	TAGTCAGTGTGGAAAATCTCTAGCA	GTCTAC	TGGAAGGGCTAATTCACTCCCAAA
73	TAGTCAGTGTGGAAAATCTCTAGCA	GTTGAC	TGGAAGGGCTAATTCACTCCCAAA
74	TAGTCAGTGTGGAAAATCTCTAGCA	GTCCAC	TGGAAGGGCTAATTCACTCCCAAA
75	TAGTCAGTGTGGAAAATCTCTAGCA	GTAAAC	TGGAAGGGCTAATTCACTCCCAAA
76	TAGTCAGTGTGGAAAATCTCTAGCA	GTGCAC	TGGAAGGGCTAATTCACTCCCAAA
77	TAGTCAGTGTGGAAAATCTCTAGCA	GTGCAC	TGGAAGGGCTAATTCACTCCCAAA
78	TAGTCAGTGTGGAAAATCTCTAGCA	GTTGAC	TGGAAGGGCTAATTCACTCCCAAA
79	TAGTCAGTGTGGAAAATCTCTAGCA	--AGAC	TGGAAGGGCTAATTCACTCCCAAA
80	TAGTCAGTGTGGAAAATCTCTAGCA	GTACCAC	TGGAAGGGCTAATTCACTCCCAAA
81	TAGTCAGTGTGGAAAATCTCTAGCA	G-GGGAC	TGGAAGGGCTAATTCACTCCCAAA
82	TAGTCAGTGTGGAAAATCTCTAGCA	G-GTAAC	TGGAAGGGCTAATTCACTCCCAAA
83	TAGTCAGTGTGGAAAATCTCTAGCA	G-GGGAC	TGGAAGGGCTAATTCACTCCCAAA
84	TAGTCAGTGTGGAAAATCTCTAGCA	G-GGAAC	TGGAAGGGCTAATTCACTCCCAAA
85	TAGTCAGTGTGGAAAATCTCTAGCA	GTCCT--	TGGAAGGGCTAATTCACTCCCAAA
86	TAGTCAGTGTGGAAAATCTCTAGCA	G-GGGAC	TGGAAGGGCTAATTCACTCCCAAA
87	TAGTCAGTGTGGAAAATCTCTAGCA	GTCCT--	TGGAAGGGCTAATTCACTCCCAAA
88	TAGTCAGTGTGGAAAATCTCTAGCA	G-GAG-C	TGGAAGGGCTAATTCACTCCCAAA
89	TAGTCAGTGTGGAAAATCTCTAGCA	GTCCAAC	TGGAAGGGCTAATTCACTCCCAAA
90	TAGTCAGTGTGGAAAATCTCTAGCA	GTATCAC	TGGAAGGGCTAATTCACTCCCAAA
91	TAGTCAGTGTGGAAAATCTCTAGCA	GTGGAAC	TGGAAGGGCTAATTCACTCCCAAA
92	TAGTCAGTGTGGAAAATCTCTA---	--CCCAC	TGGAAGGGCTAATTCACTCCCAAA
93	TAGTCAGTGTGGAAAATCTCTA---	--CCCAC	TGGAAGGGCTAATTCACTCCCAAA
94	TAGTCAGTGTGGAAAATCTCTAGCA	--ACGAC	TGGAAGGGCTAATTCACTCCCAAA
95	TAGTCAGTGTGGAAAATCTCTA---	--CCCAC	TGGAAGGGCTAATTCACTCCCAAA
96	TAGTCAGTGTGGAAAATCTCTAGCA	GTGGGAC	TGGAAGGGCTAATTCACTCCCAAA
97	TAGTCAGTGTGGAAAATCTCTAGCA	GTCGCCAC	TGGAAGGGCTAATTCACTCCCAAA
98	TAGTCAGTGTGGAAAATCTCTAGCA	G-AGCCAC	TGGAAGGGCTAATTCACTCCCAAA
99	TAGTCAGTGTGGAAAATCTCTAGCA	GTGGCAAC	TGGAAGGGCTAATTCACTCCCAAA
100	TAGTCAGTGTGGAAAATCTCTAGCA	GTCCCTAC	TGGAAGGGCTAATTCACTCCCAAA
101	TAGTCAGTGTGGAAAATCTCTAGCA	G-AGCCAC	TGGAAGGGCTAATTCACTCCCAAA
102	TAGTCAGTGTGGAAAATCTCTAGCA	GTGGGC--	TGGAAGGGCTAATTCACTCCCAAA
103	TAGTCAGTGTGGAAAATCTCTAGCA	G-GGGA--	TGGAAGGGCTAATTCACTCCCAAA
104	TAGTCAGTGTGGAAAATCT------	--AAAGAC	TGGAAGGGCTAATTCACTCCCAAA
105	TAGTCAGTGTGGAAAATCTCTAGCA	--AGCCAC	TGGAAGGGCTAATTCACTCCCAAA
106	TAGTCAGTGTGGAAAATCTCTAGCA	G-GGTC--	--GAAGGGCTAATTCACTCCCAAA
107	TAGTCAGTGTGGAAAATCTCTAGCA	GTGGGGAC	TGGAAGGGCTAATTCACTCCCAAA
108	TAGTCAGTGTGGA------------	--GGCC--	TGGAAGGGCTAATTCACTCCCAAA
109	TAGTCAGTGTGGAAAATCTCTAGCA	GTGGGGAC	TGGAAGGGCTAATTCACTCCCAAA
110	TAGTCAGTGTGGAAAATCTCTAGCA	G-GGGGGAC	TGGAAGGGCTAATTCACTCCCAAA
111	TAGTCAGTGTGGAA-----------	--CCCCCAC	TGGAAGGGCTAATTCACTCCCAAA
112	TAGTCAGTGTGGAAAATCTCTAGCA	GTAAGCCAC	TGGAAGGGCTAATTCACTCCCAAA
113	TAGTCAGTGTGGAAAATCTCTAGCA	G-GGGCCAC	TGGAAGGGCTAATTCACTCCCAAA
114	TAGTCAGTGTGGAAAATCTCTAGCA	G-ACCTAAC	TGGAAGGGCTAATTCACTCCCAAA
115	TAGTCAGTGTGGAAAATCTCTAGCA	G-GGGGGAC	---AAGGGCTAATTCACTCCCAAA
116	TAGTCAGTGTGGAAAATCTCTAGC-	--GGGGGAC	TGGAAGGGCTAATTCACTCCCAAA
117	TAGTCAGTGTGGAAAATCTCTAGCA	--CTGGA--	------GGCTAATTCACTCCCAAA
118	TAGTCAGTGTGGAAAATCTCTAGCA	GTCCCCC--	---AAGGGCTAATTCACTCCCAAA
119	TAGTCAGTGTGGAAAATCTCTAGC-	--GGGGGAC	TGGAAGGGCTAATTCACTCCCAAA
120	TAGTCAGTGTGGAAAATCTCTAGC-	--GGGGGAC	TGGAAGGGCTAATTCACTCCCAAA
121	TAGTCAGTGTGGAAAATCTCTAGCA	GTGAACCAC	TGGAAGGGCTAATTCACTCCCAAA
122	TAGTCAGTGTGGAAAATCTCTAGCA	GTTTCCCAC	TGGAAGGGCTAATTCACTCCCAAA
123	TAGTCAGTGTGGAAAATCTCTAGCA	GTCTTCA--	TGGAAGGGCTAATTCACTCCCAAA
124	TAGTCAGTGTGGAAAATCTCTAGCA	GTGGCGCC--	-GGAAGGGCTAATTCACTCCCAAA
125	TAGTCAGTGTGGAAAATCTCTAGCA	G-AGGAATAC	TGGAAGGGCTAATTCACTCCCAAA
126	TAGTCAGTGTGGAAAA---------	--ACGCCCAC	TGGAAGGGCTAATTCACTCCCAAA
127	TAGTCAGTGTGGAAAATCTCTAG--	--GGGGTCAC	TGGAAGGGCTAATTCACTCCCAAA
128	TAGTCAGTGTGGAAAATC-------	--CTCCCT--	---------TAATTCACTCCCAAA
129	TAGTCAGTGTGGAAAATCTCTAGCA	G-AGGAATAC	TGGAAGGGCTAATTCACTCCCAAA
130	TAGTCAGTGTGGAAAATCTCTAGCA	GTGGTCCAAC	TGGAAGGGCTAATTCACTCCCAAA
131	TAGTCAGTGTGGAAAATCTCTAGCA	GTGGGGTCAC	TGGAAGGGCTAATTCACTCCCAAA
132	TAGTCAGTGTGGAAAATCTCTAGCA	GTCCTCGAAC	TGGAAGGGCTAATTCACTCCCAAA
133	TAGTCAGTGTGGAAAATCTCTAGC-	--TCCGAGG--	-----GGGCTAATTCACTCCCAAA
134	TAGTCAGTGTGGAAAATCTCTAGCA	GTTCGGGCGGAC	TGGAAGGGCTAATTCACTCCCAAA
135	TAGTCAGTGTGGAAAATCTCTAGCA	GTTAGAGCATA--	---AAGGGCTAATTCACTCCCAAA
136	TAGTCAGTGTGGAAAATCTCTAGCA	GTAAAAGGGGGGAC	TGGAAGGGCTAATTCACTCCCAAA
137	TAGTCAGTGTGGAAAATCTCTAGCA	GTAAAAGGGGGGAC	TGGAAGGGCTAATTCACTCCCAAA
138	TAGTCAGTGTGGAAAATCTCTA---	--ACGCGAAGGG--	-----GGGCTAATTCACTCCCAAA
139	TAGTCAGTGTGGAAAATCTCTAGC-	--CCATCGTTGACAC	TGGAAGGGCTAATTCACTCCCAAA
140	TAGTCAGTGTGGAAAATC-------	--CCCCAGGGGCCAC	TGGAAGGGCTAATTCACTCCCAAA
141	TAGTCAGTGTGGAAAATCTCTAGCA	--TCCTTCCCTCC-C	TGGAAGGGCTAATTCACTCCCAAA
142	TAGTCAGTGTGGAAAATCTCTAGCA	GTGGCCTGTTTTCAC	TGGAAGGGCTAATTCACTCCCAAA
143	TAGTCAGTGTGGAAAATCTCTAGCA	--TCCTTCCCTCCC--	TGGAAGGGCTAATTCACTCCCAAA
144	TAGTCAGTGTGGAAAATCTCTAGCA	G-GGGTCCCCGAACG--	------------TTCACTCCCAAA
145	TAGTCAGTGTGGAAAATCTCTAGCA	GTGGCGCCCGAACAG--	TGGAAGGGCTAATTCACTCCCAAA
146	TAGTCAGTGTGGAAAATCTCTAGCA	GTGGCGCCCGAACAGGGAAC	TGGAAGGGCTAATTCACTCCCAAA
147	TAGTCAGTGTGGAAAATCTCTAGCA	GTGGCGCCCGAACAGGGACAC	TGGAAGGGCTAATTCACTCCCAAA
148	TAGTCAGTGTGGAAAATCTCTAGCA	GTGGCGCCCGAACAGGGACAC	TGGAAGGGCTAATTCACTCCCAAA
149	TAGTCAGTGTGGAAAATCTCTAGCA	GTTGGCGCCCGAACAGGGAAC	TGGAAGGGCTAATTCACTCCCAAA
150	TAGTCAGTGTGGAAAATCTCTAGCA	GTTGGTGTTTCCGCCCGGTTAC	TGGAAGGGCTAATTCACTCCCAAA
151	TAGTCAGTGTGGAAAATCTCTAGCA	GTTGGTGTTTCCGCGCGGGTAC	TGGAAGGGCTAATTCACTCCCAAA
152	TAGTCAGTGTGGAAAATCTCTAGCA	GTGGCGCCCACCCAGGGACGCCAAC	TGGAAGGGCTAATTCACTCCCAAA
153	TAGTCAGTGTGGAAAATCTCTAGCA	GTCATCTAGAAAGAAAAGGGGGGAC	TGGAAGGGCTAATTCACTCCCAAA
154	TAGTCAGTGTGGAAAATCTCTAGCA	GTGATTTTAAAAGAAAAGGGGGGAC	TGGAAGGGCTAATTCACTCCCAAA
155	TAGTCAGTGTGGAAAA---------	--ACCTCCCCAGCATCTCCACCA--	-GGAAGGGCTAATTCACTCCCAAA
156	TAGTCAGTGTGGAAAA---------	--ACCTCCCCAGCATCTCCACCA--	-GGAAGGGCTAATTCACTCCCAAA
157	TAGTCAGTGTGGAAAA---------	--ACCTCCCCAGCATCTCCACCA--	-GGAAGGGCTAATTCACTCCCAAA
158	TAGTCAGTGTGGAAAATCTCTAGCA	GTTACCATTTTTAAAAGAAAAGGAGGGAC	TGGAAGGGCTAATTCACTCCCAAA
159	TAGTCAGTGTGGAAAATCTCTAGCA	GTTAGCCAATTTTAAAAGAAAAGGGGGGAC	TGGAAGGGCTAATTCACTCCCAAA
160	TAGTCAGTGTGGAAAATCTCTAGCA	GTGGCGCCCGAACAGGGACTTGAAAGCGAAAC	TGGAAGGGCTAATTCACTCCCAAA
161	TAGTCAGTGTGGAAAATCTCTAGCA	GTGGCGCCTGAACAGGGACTTGAAAGCGAAAC	TGGAAGGGCTAATTCACTCCCAAA
162	TAGTCAGTGTGGAAAATCTCTAGCA	GTTCTTAGCCACTTTTTAAAAGAAAAGGGGGGAC	TGGAAGGGCTAATTCACTCCCAAA
163	TAGTCAGTGTGGAAAATCTCTAGCA	GTTGGCGTCCTTGGGATCCCAATTCTGACACCAGAC	TGGAAGGGCTAATTCACTCCCAAA
164	TAGTCAGTGTGGAAAATCTCTAGCA	G-GCTCTTAGCCACTTTTTAAAAGAAAAGGGGGGAC	TGGAAGGGCTAATTCACTCCCAAA
165	TAGTCAGTGTGGAAAATCTCTAGCA	GTGGCGCCTGAACAGGGACTTGAAAGCGAAAGTAAA--	TGGAAGGGCTAATTCACTCCCAAA
166	TAGTCAGTGTGGAAAATCTCTAGCA	--ACAAGGCAGCTGTAGATCTTAGCCACTTTTTAAAAGAAAAGGGGGGAC	TGGAAGGGCTAATTCACTCCCAAA
